# New human ATM variants are able to regain ATM functions in ataxia telangiectasia disease

**DOI:** 10.1007/s00018-022-04625-3

**Published:** 2022-11-23

**Authors:** Anastasia Ricci, Federica Biancucci, Gianluca Morganti, Mauro Magnani, Michele Menotta

**Affiliations:** Department of Biomolecular Sciences, University of Urbino “Carlo Bo”, Via Saffi 2, 61029 Urbino, Italy

**Keywords:** Foci number, Cell cycle checkpoint, ATM/ATR crosstalk, Oxidative stress

## Abstract

Ataxia telangiectasia is a rare neurodegenerative disease caused by biallelic mutations in the ataxia telangiectasia mutated gene. No cure is currently available for these patients but positive effects on neurologic features in AT patients have been achieved by dexamethasone administration through autologous erythrocytes (EryDex) in phase II and phase III clinical trials, leading us to explore the molecular mechanisms behind the drug action. During these investigations, new ATM variants, which originated from alternative splicing of ATM messenger, were discovered, and detected in vivo in the blood of AT patients treated with EryDex. Some of the new ATM variants, alongside an in silico designed one, were characterized and examined in AT fibroblast cell lines. ATM variants were capable of rescuing ATM activity in AT cells, particularly in the nuclear role of DNA DSBs recognition and repair, and in the cytoplasmic role of modulating autophagy, antioxidant capacity and mitochondria functionality, all of the features that are compromised in AT but essential for neuron survival. These outcomes are triggered by the kinase and further functional domains of the tested ATM variants, that are useful for restoring cellular functionality. The in silico designed ATM variant eliciting most of the functionality recover may be exploited in gene therapy or gene delivery for the treatment of AT patients.

**Supplementary Information:**

The online version contains supplementary material available at 10.1007/s00018-022-04625-3.

## Introduction

Ataxia telangiectasia (AT) is a rare autosomal recessive neurodegenerative disease with a prevalence between 1 in 40,000 and 1 in 100,000 live births worldwide, caused by biallelic mutations in the *Ataxia Telangiectasia Mutated* (*ATM*) gene (Chr 11q22.3–23.1). Over 400 ATM mutations have been identified on the *ATM* gene, most of which are non-sense mutations, giving rise to non-functional proteins [1, 2]. *ATM* gene codes for the protein of the same name ATM, a member of the phosphatidylinositol 3-kinase-related kinases (PIKK) family [3, 4], with multiple substrates. This protein is activated into an active monomer by auto-transphosphorylation of Ser1981, Ser367 and Ser1893 [5]. It is implicated in the nuclear role of DNA double-strand breaks (DSBs) repair, phosphorylating various substrates to start the DNA repair signaling cascade and preventing DNA damage accumulation. Beyond its nuclear role, ATM was found to be triggered by reactive oxygen species (ROS) in the cytoplasm as an active dimer, formed through the disulfide bond of Cysteines at position 2991 in the FRAP-ATM-TRRAP (FAT)-C-terminal (FATC) domain, in an independent manner from DNA DSBs activation [6]. Currently, several studies correlated ATM dimer activation to ROS production, suggesting new roles of ATM in regulating oxidative stress, autophagy, mitophagy and protein homeostasis [7–13]. All these ATM pleiotropic effects are still under investigation, as their contribution could explain the neurodegenerative process that occurs in AT patients. The severity of the disease indeed, depends on the residual presence and function of the ATM protein leading to the classical AT phenotype when the ATM protein is completely absent, and to a milder phenotype if a residual ATM function is present [14]. Classically, AT patients show a complex phenotype, characterized primarily by a progressive cerebellar ataxia with loss of Purkinje cells and oculocutaneous telangiectasias. Further features include sensitivity to ionizing radiation, immunodeficiency, high susceptibility to the development of tumors (lymphoma and leukemia), infections (respiratory infections), and endocrine abnormalities [15–18]. Unfortunately, no cure is currently available for these patients, but only palliative treatments to alleviate their symptoms. In the last decade, it has been occasionally found that glucocorticoid treatment improves the neurological symptoms of AT patients [19–23], however, the molecular mechanism behind the drug action is still unknown. The discovery of a shorter transcript derived from a non-canonical splicing of the native ATM messenger, named ATMdexa1, induced by dexamethasone action in vitro, that can be translated into a functional protein, named ‘miniATM’ [24], shed light on the positive effect of the drug. ATMdexa1 and further new ATM variants originating from canonical (exons 3-52, 4-53 and 2-52) and non-canonical (short direct repeat: 3-52 and 4-51) splicing were also found in the blood of AT patients undergoing glucocorticoids treatment through autologous erythrocytes in a phase II clinical trial [19, 20]. The expression of said variants correlated with an improvement in neurological symptoms of AT patients [25]. In this regard, prompted by the likely beneficial function of ‘miniATM’, we have investigated the potential role of the new selected ATM variants (named: ATM 3-52, ATM 4-53) when expressed in AT fibroblasts by the lentiviral system. A third ATM variant, named ATM SINT, was in silico designed and characterized, with the addition of functional domains. The resulting proteins have more domains than miniATM itself, and we were able to demonstrate that these ATM variants were useful in vitro in providing some compromised functions of the native ATM, especially concerning its central role in preserving genome integrity and preventing oxidative stress, both of which are critical for neuronal survival. The regained ATM function, despite not being a complete recovery, gave new hope for the development of innovative therapies for both gene therapy approaches and/or non-viral gene delivery, to treat AT patients. The reduced cDNA size compared to the huge size of the *ATM* gene means that classical gene therapy is now a possibility. In fact, the transduction of fibroblast cells with ATM variants through a lentiviral system, here described, achieved a high transduction efficiency (almost 100%), and therefore could be more advantageous for vectors which have been approved for gene therapy. These combined results demonstrate the potential advantageous roles of ATM variants in becoming a possible treatment for AT patients.

## Materials and methods

### Cell culture and treatments

Fibroblasts WT AG09429 (*Atm*^+/+^) and AT GM00648 (*Atm*^−/−^) from Coriell Institute (Camden, NJ, USA) were used as a cellular model. The hTERT antigen cell immortalization Kit (Alstem Cell Advancements) was used to immortalize the cells. The selected AT GM00648 hTERT (AT 648 hT) and WT AG09429 hTERT (WT hT) were grown in MEM (Eagle formulation). The medium was supplemented with 2 mmoL/L l-glutamine, 100 U/mL penicillin, and 0.1 mg/mL streptomycin (Sigma Aldrich), 15% fetal bovine serum (Thermo Fisher Scientific) and 10 mM glucose. All cells were incubated at 37 °C with 5% CO_2_. Human embryonic kidney (HEK) 293 T cells (ATCC^®^ CRL-3216™), used for transfection in lentiviral particles production, were grown in D-MEM (Eagle formulation). The medium was supplemented with 2 mmoL/L l-glutamine, 100 U/mL penicillin, and 0.1 mg/mL streptomycin (Sigma Aldrich), and 10% fetal bovine serum (MERK).

### Lentiviral vector construction and production

ATM 3-52, ATM 4-53, ATM SINT and miniATM cDNAs (the sequences of the tested variants are reported in Fig. S1) were inserted into pLenti-C-Myc-DDK-IRES-neo tagged cloning vector with double selection: chloramphenicol for *E. coli* selection and neomycin for mammalian cell selection. WT hT and AT 648 hT untransduced cells were used as reference and negative control, respectively. Viral particles were produced by co-transfecting HEK 293T cells in 24-well plates (1.2 × 10^5^/well) with cloned ATM variants using MegaTran1.0 Transfection Reagent as reported by the Lenti-vpak Lentiviral Packaging Kit (OriGene). Viral particles were collected and concentrated according to the method reported by Miller et al. [26].

### Transduction of cells

4 × 10^4^ AT 648 hT cells per well were seeded in 24-well plates and after 24 h, viral particles were added to cells in the presence of 5 µg/mL of polybrene (MERK). Clones’ selection was performed as indicated by the Lenti-vpak Lentiviral Packaging Kit supplier. AT 648 hT transduced cell lines were called TD 3-52 for ATM 3-52, TD 4-53 for ATM 4-53, TD SINT for ATM SINT, and TD miniATM for miniATM. UTD is referred to AT 648 hT untransduced cells.

### DSBs induction by bleomycin

To determine whether the ATM constructs were able to counteract the DNA damage response, WT hT and AT 648 hT transduced and untransduced cells were treated with bleomycin at a final concentration of 8 µg/mL, equivalent to exposure of about 3.2 Gy γ-radiation [27]. Cells underwent three types of treatment: placebo solution, 3 h of bleomycin treatment, and a subsequent 24-h incubation in drug-free culture medium.

ATR crosstalk activity was tested by using the ATR kinase inhibitor VE-821 [28]. Before the addition of bleomycin, WT hT and AT 648 hT transduced and untransduced cells were exposed to 10 µM of VE-821 for 1 h, and then cells were co-exposed to 8 µg/mL of bleomycin for 3 h in presence of the ATR inhibitor [29]. Cells treated with VE-821 were compared to cells treated with bleomycin alone and with the only vehicle (DMSO).

### Western blotting

Total proteins were extracted using the Protein Extraction Reagent Type 4 (P4, Sigma Aldrich). Cells were sonicated with 10 pulses of 15 s at 45 W Labsonic 1510 Sonicator (Braun) and clarified by centrifugation for 10 min at 10,000 RCF. Protein concentration was determined by the Bio-Rad Protein Assay, based on Bradford’s method. Twenty micrograms of proteins were separated by SDS-PAGE (Novex TrisGlycine gels) according to the Laemmli protocol [30] and then transferred to nitrocellulose (0.22 µm, Bio-Rad) or LF PVDF (0.45 µm, Bio-Rad) by wet transfer and Towbin blotting buffer (50 mM Tris, 150 mM NaCl, 20%v/v methanol). Membranes were probed with the primary antibodies diluted in 5% w/v non-fat dry milk or 5% BSA in TBS-T. The primary antibodies used in this study were: anti-phospho H2AX Ser139 (GeneTex and Cell Signaling Technology, CST), anti-phospho p53 Ser15 (CST), anti-p53 (Santa Cruz Biotechnology, SCBT), anti-phospho CHK2 Thr68 (CST and AB clonal), anti-phospho ATR Ser428 (CST), anti-ATR (Bethyl), anti-LC3B (CST), anti-SQSTM1/p62 (CST), anti-calreticulin (CST), anti-pATM Ser1981 (CST), anti-ATM (1B10 Abnova). The utilized secondary antibodies were anti-rabbit and anti-mouse HRP coniugated (BIORAD), anti-rabbit StarBrightBlue700 (Biorad) and Alexa Fluor 790 (Thermo Fisher Scientific), and anti-mouse Alexa Fluor 680 (Thermo Fisher Scientific). Immunoreactive bands were recorded using the enhanced chemiluminescence (Advansta) or fluorescence acquisition by ChemiDoc Touch Imaging System (Bio-Rad). The whole lane normalization (WLN) strategy was adopted in all western blot analyses using a trihalo compound for protein visualization [31–33]. Acquired images were analyzed by Image Lab software 5.2.1 (Bio-Rad) [34].

### Indirect immunofluorescence microscopy

1 × 10^5^ cells per well were grown on Lab-Tek II chamber slide (Nunc) 8-well slides upon reaching 70–80% of confluence. After bleomycin treatment for γH2AX detection, and for HDAC4 detection, cells were fixed with 4% formaldehyde for 10 min and then with 100% cold methanol for 10 min. They were subsequently permeabilized with 0.5% NP-40 in PBS for another 10 min. After performing the blocking procedure for 1 h at room temperature, primary antibodies were applied in 0.1% Triton X100, 1% BSA in PBS overnight at 4 °C. The following antibodies were used: anti-phospho H2AX Ser139 (GeneTex and Cell Signaling Technology) and anti-HDAC4 (Cell Signaling Technology and Thermo Fisher Scientific). The following day, slides were incubated with secondary anti-mouse TRITC-conjugated antibody (Sigma-Aldrich) or anti-rabbit FITC-conjugated antibody (Sigma-Aldrich) in 0.1% Triton X100, 1% BSA in PBS for 1 h at 37 °C. After washing procedures, DNA was stained with 4′,6-diamidino-2-phenylindole (DAPI) at a final concentration of 0.2 µg/mL. Washed slides were mounted and embedded with ProLong Antifade (Thermo Fisher Scientific). Slides were observed by Olympus IX51, and the images were acquired by ToupCam camera (ToupTek Europe). Image analyses were performed by ImageJ (NIH) (developed at the US National Institutes of Health and available on the Internet at http://rsb.info.nih.gov/nih-image), and H2AX foci numbers were indirectly calculated (after system calibration) by nuclear signal skewness data.

### DCFH-DA cellular assay

Cells were seeded in black 96-well plates (6000/well), and 24 h later, intracellular ROS levels were examined using a non-fluorescent agent 2′,7′-dichlorofluorescin diacetate (DCFH-DA, Sigma-Aldrich, Milan, Italy), as previously reported by Benedetti et al. [35] with slight modifications. Cells were incubated with DCFH-DA (5 µM) at 37 °C for 30 min, then the excess probe was removed by washing cells with PBS. DCF oxidation kinetic was detected both at basal condition for 30 min, and after the addition of H_2_O_2_ (100 µM) for additional 30 min, monitoring the fluorescence signal.

The fluorescence emission of the probe was measured at 520 nm upon excitation at 485 nm in a FluoStar Optima spectrofluorimeter (BMG Labtech, Offenburg, 223 Germany).

### Mitotracker Red CMX-ROS assay

Mitochondrial membrane potential was detected by staining live-cells using the Mitotracker Red CMX-ROS [36]. Cells were seeded in black 96-well plates (6000/well) and 24 h later, cells were incubated with the probe (100 nM) diluted in serum-free medium for 18 min (previously verified as non-saturating endpoint), monitoring the dye entry kinetic measuring the emission fluorescence of the probe at 612 nm upon excitation at 584 nm by a FluoStar Optima spectrofluorimeter (BMG Labtech, Offenburg, 223 Germany).

### Mitochondrial DNA quantification in the cytoplasm

The release of mtDNA in the cytoplasm has been performed as reported by Yang et al. [37]. The amount of mtDNA was quantified performing a quantitative PCR by using primers amplifying NADH-ubiquinone oxidoreductase chain 1 (ND1) DNA [38], a specific mitochondrial gene from 5 ng of the cytoplasmic DNA fraction. The Proteasome 20S Subunit Beta 5 (PSMB5) nuclear reference gene was quantified by using the primers: forward 5′-ACGTGGACAGTGAAGGGAAC–3′ and reverse 5′-CTGCTCCACTTCCAGGTCAT-3′, from 2 ng of the nuclear DNA fraction. PCR reactions were performed on a QuantStudio™ 5 Real-Time PCR System and amplification plots were analyzed using the QuantStudio™ sequence detection system (Applied Biosystems) and the relative expression data were calculated by the 1/2^ΔCt^ method.

### NAD^+^ assay quantification

For the NAD^+^ quantitation, metabolites were extracted from cell pellets (6 × 10^6^) in ice-cold lysis buffer 6:4 MeOH:ACN, 0.1% formic acid; samples were vortexed and incubated at − 20 °C for sample deproteinization. After centrifugation the supernatants were collected and freeze-dried.

The samples were resuspended with 50:30:20 MeOH:ACN:H_2_O with 0.1% formic acid for the injection in an UHPLC Vanquish system (Thermo Fisher scientific) coupled to an orbitrap Exploris 240 mass spectrometer. The metabolites separation was performed by an Accucore 150 amide HILIC column (held at 60 °C) and the mobile phases consisted in the phase A, water with 0.1% formic acid and B acetonitrile with 0.1% formic acid both containing 5 mM ammonium formiate. Elution gradient was 99% of B up to 3 min, 1%B in 11 min, 1%B for 4 min 99%B in 0.2 min and 99%B up to 22 min. The mass spectrometer, equipped with H-ESI source was operated in positive mode with a scan range 80–800 *m*/*z* in DDA manner. Deep scanning strategy was adopted by AcquireX and NAD+ identification and quantitation was performed by Compound Discoverer 3.2 (Thermo Fischer scientific).

### Statistical analysis

GraphPad Prism was used for statistical analyses and graph generation. Statistical tests were chosen according to sample size and variance homogeneity. The statistical tests used for the comparison of more than two groups were: Friedman test followed by Dunn’s test for WB experiments and NAD^+^ metabolite assay; Kruskal–Wallis test followed by Dunn’s test for IF, DCF and mtDNA assays; Welch’s ANOVA test for foci analyses and Mitotracker assay. Mann–Whitney test was used to compare two unpaired samples, while *t* test for unpaired measures was applied when data were normally distributed. Means or medians were considered statistically different when *p* ≤ 0.05.

## Results

### ATM variants description

Several ATM variants have been identified in vivo in the blood of AT patients treated with EryDex in a phase II Clinical trial, originated from canonical and non-canonical splicing of the native ATM messenger [25]. All these natural variants share the miniATM sequence plus additional domains of the full-length ATM and could provide an improved restoration of the AT phenotype. Among them, ATM 3-52 has been the most frequent ATM variant found in patients (32%, data not shown), and its translation start 714 bp upstream than miniATM starting codon, even though not the native one. Meanwhile, ATM 4-53, can be translated from the native starting codon. This confers to the translated protein the Telomere-length maintenance and DNA damage repair (TAN) domain, useful for the DNA damage response. A third variant, named as ATM SINT has been designed in silico, adding functional domains compared to ATM 3-52 and ATM 4-53, to evaluate whether it could be able to regain most of the nuclear and cytoplasmic ATM functions.

### Lentiviral constructs assay

The AT 648 hT fibroblasts, that were transduced by using the lentiviral system, were tested for the expression of the cloned ATM variants.

**Fig. 1:**
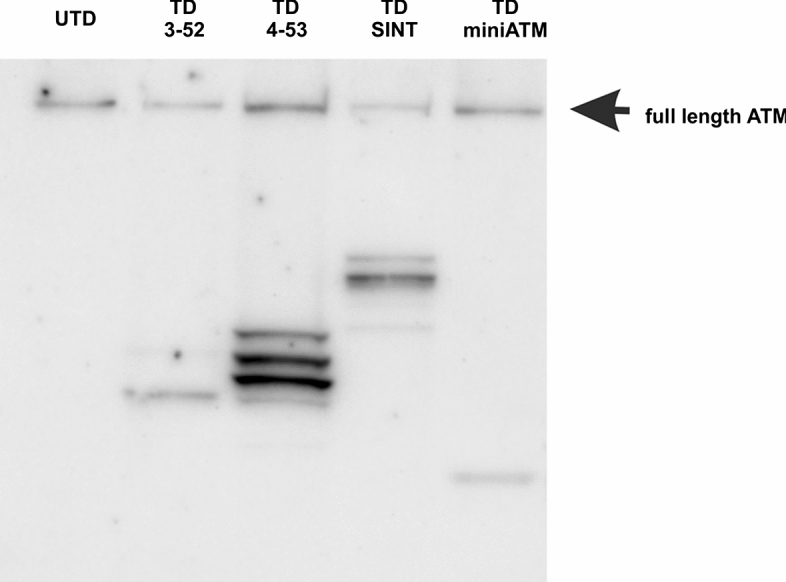
WB of UTD and AT 648 hT fibroblasts expressing ATM 3-52, ATM 4-53, ATM SINT, miniATM. The arrow indicates the native mutated full-length ATM

The expression was verified by Western Blotting (WB) using an anti-ATM antibody that targets the C-terminal domain of ATM (clone 1B10) and Fig. 1 shows TD 3-52, TD 4-53, TD SINT and TD miniATM. The UTD was also probed as control. The native mutated full-length ATM was also noticed in all the tested cell lines.

### ATM variants activity in DSBs

**Fig. 2:**
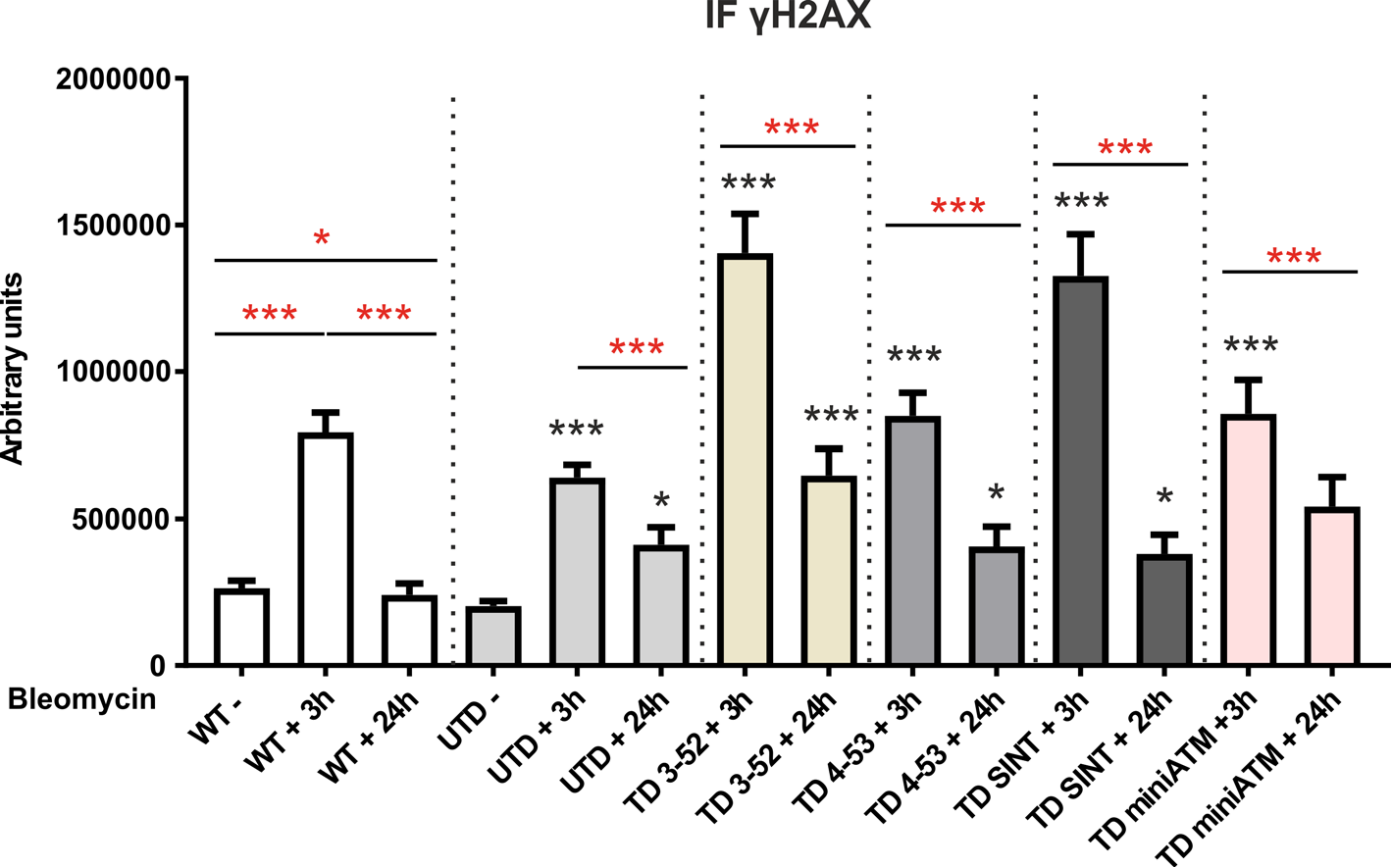
AT 648 hT transduced cells overcome ATM functions in recognition and repair of DSB sites. Quantification of γH2AX IF experiments of WT hT and AT 648 hT transduced and untransduced cells treated with bleomycin for 3 h and then kept in recovery course for 24 h. In WT hT cells, H2AX phosphorylation enhanced at 3 h post drug, and underwent a reduction after 24 h of recovery. All TD cells showed a higher H2AX phosphorylation after 3 h of bleomycin compared to UTD at basal condition, and a dephosphorylation at 24 h post drug compared to their treated counterparts. Unexpectedly, also UTD followed this pattern, revealing a probable crosstalk between ATM/ATR. At least 300 nuclei from three independent experiments were counted for data processing. Red asterisks refer to intra sample comparison, while the black ones refer to statistical comparison with the control cell line UTD (Kruskal Wallis followed by Dunn’s test). Graphs show mean with SEM

**Fig. 3:**
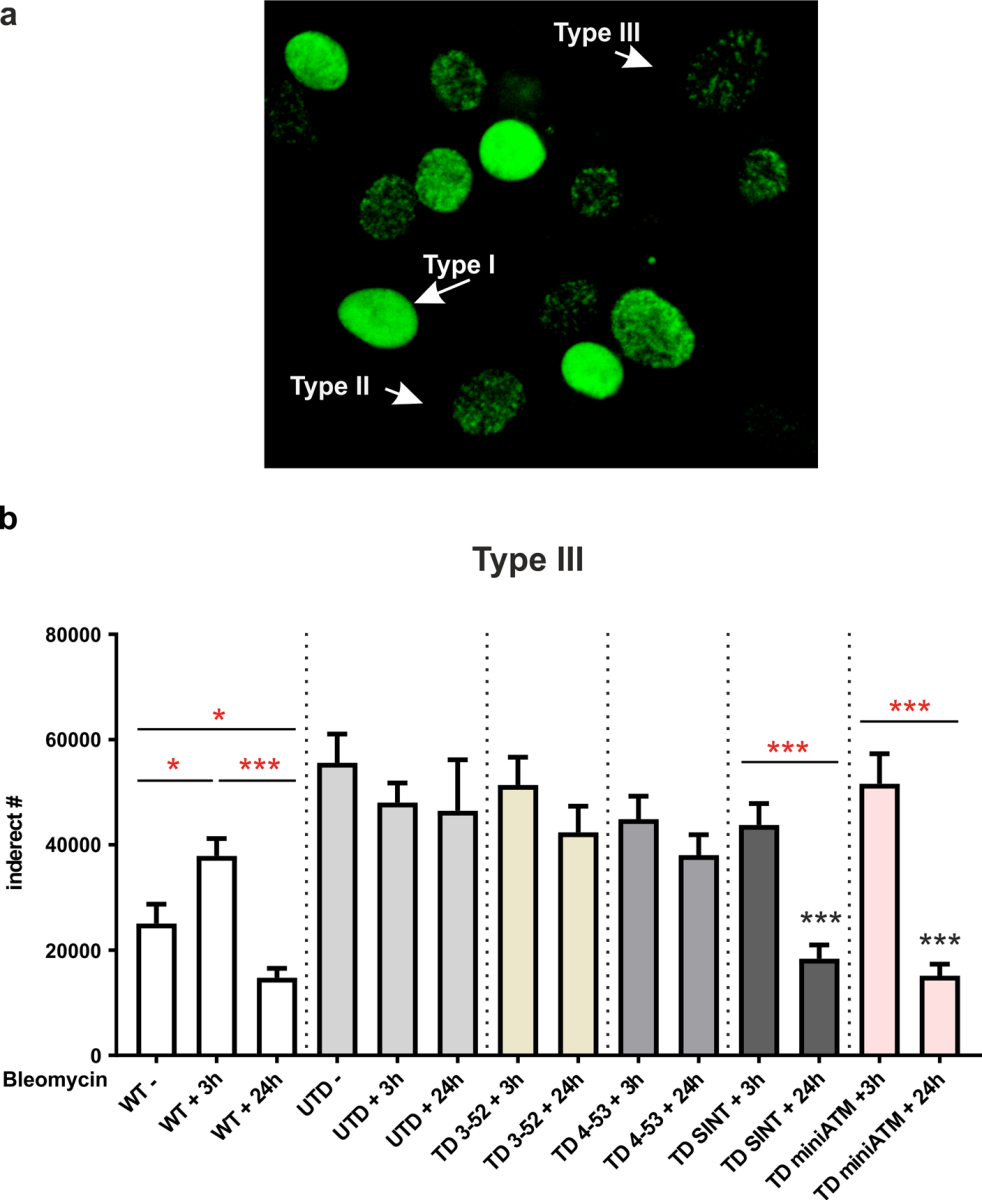
ATM SINT is the most efficient ATM variant in repairing lesions, as confirmed by the reduced type III foci number 24 h post drug. **a** Example of γH2AX foci staining pattern. **b** Quantitation of type III foci in all samples. The type III foci are suitable to DNA repair efficiency assessment, implying that the γH2AX foci decrement at 24 h indicates successful DNA repair. Type III foci statistically increased and decreased after bleomycin treatment and 24 h post drug, respectively, in WT hT cells. TD 3-52, TD 4-53 did not present a statistically decrease of type III foci, while only TD SINT and TD miniATM presented a significant decrease of foci number during the recovery. In UTD, bleomycin treatment did not affect the composition of type III foci. In fact, they showed the same number of foci in all three treatment conditions, indicating a persistence of unrepaired lesions and confirming a different pathway on H2AX phosphorylation in these cells. At least 300 nuclei from three independent experiments were counted for data processing. Red asterisks refer to intra sample comparison, while the black ones show the statistical comparison with the control cell line UTD (Welch’s ANOVA test). Graphs show mean with SEM

The biochemical properties of the investigated ATM variants were evaluated by testing the canonical ATM targets after bleomycin treatment, that causes double strand breaks in DNA [39]. Among its downstream target, phosphorylation of histone H2AX at Ser139 (γH2AX) is considered one of the first events after DSBs [40], forming nuclear foci easily detectable at the break sites, which is necessary for the identification and repair of DSBs [41–43]. Accordingly, γH2AX was detected by indirect immunofluorescence (IF) assay with different approaches to quantify the DNA damage: measuring fluorescence intensity (Figs. 2 and S2) and analyzing the quality of foci distribution (Fig. 3) [44, 45]. As reported in Figs. 2 and S2, in WT hT cells and in all TD cells there was an increase in γH2AX content after 3 h drug treatment compared to untreated UTD, indicating that the expressed ATM variants were correctly activated in response to DNA damage, while a significant decrement of γH2AX was observable 24 h post treatment. Surprisingly, also the analysis of γH2AX in UTD showed the same pattern observed in TD cells. H2AX phosphorylation assay performed by WB showed the same behavior observed by IF for all treated cells, as reported in Fig. S3.

Once established that ATM variants were properly activated upon bleomycin treatment, we proceeded to estimate the quality of foci distribution, as reported by Lu et al. [45], pointing out the priority on evaluating the foci number rather than signal intensity to monitor DSBs repair process. We in turn allocated the fluorescence signal of H2AX phosphorylation into three categories as a function of signal skewness (Fig. 3a): type I: skew < 0.05 (pan-nuclear staining), type II: 0.05 ≤ skew ≤ 2 (indistinct foci) and type III: skew > 2 (distinct countable foci), and subsequently, we obtained foci quantification from the type III stain pattern (Fig. 3b). DNA lesions correction depends on foci number reduction. Figure S4 shows staining types of each tested cell line after the applied treatment, and we observed that all the AT 648 hT transduced cells presented an increment of type I stain pattern after 3 h of drug treatment compared to the untreated UTD, and an increment of type III stain pattern 24 h post drug compared to their treated counterparts. In contrast, the analysis of foci number (Fig. 3b) revealed that TD SINT are the only ones efficient in repairing lesions, as demonstrated by the reduced number of type III foci over a period of 24 h. On the contrary, TD 3-52 and TD 4-53 were not able to statistically decrease the foci number in the same lapse of time. TD MiniATM exhibited a significant diminished number of foci at 24 h post treatment, but they are the least capable in phosphorylating H2AX, compared to other TD cells (Fig. 2, Welch test). Treated WT hT cell lines consistently increased type I over a period of 3 h (Fig. S4), as ATM correctly phosphorylated H2AX and presented a significant loss of foci number 24 h post bleomycin treatment (Fig. 3b), suggesting that most foci are solving, and the lesions are reduced. Additionally, a slight increase in type I staining pattern after 3 h of bleomycin treatment was also noticed in UTD (Fig. S4), but high numbers of foci remained, independently of bleomycin treatment (Fig. 3b). These data could indicate the presence of a crosstalk between ATM and ATR in UTD.

### UTD are influenced by ATR

**Fig. 4:**
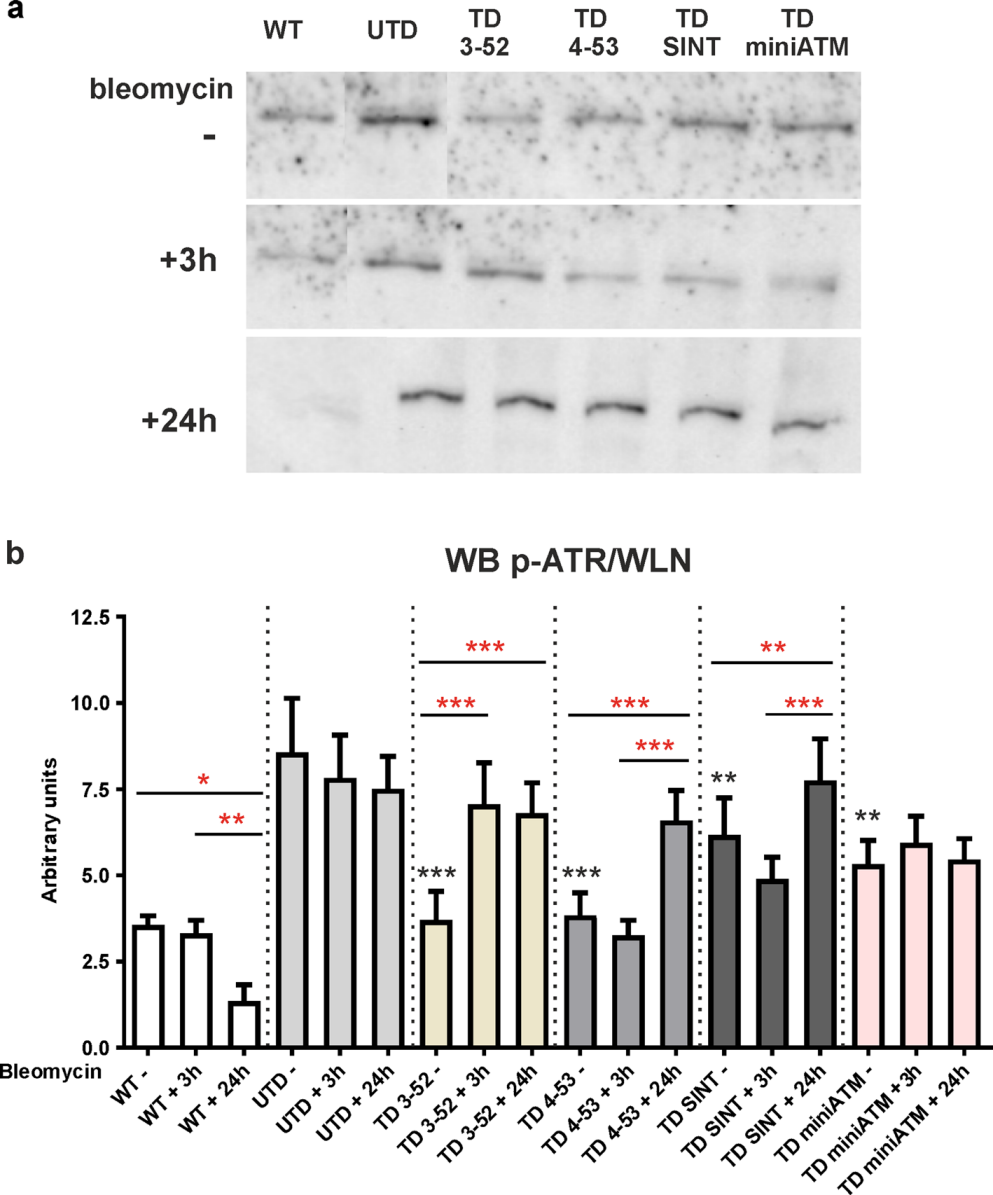
γH2AX is influenced by ATR in UTD. **a** Typical WB representative image and **b** quantification of immunoreactive bands of p-ATR/WLN ratio in all the tested cells treated with bleomycin for 3 h and then kept in recovery for 24 h. p-ATR was tested to evaluate the replication stress in UTD, revealing an elevated quantity of p-ATR in these cells, irrespective of bleomycin treatment. At basal condition, in all TD cells p-ATR is lower than UTD, suggesting a reduction of replication stress in these cells. After 3 h of bleomycin treatment, p-ATR amount enhanced only in TD 3-52, while after 24 h of recovery it increased in all TD cells (except for TD miniATM). TD miniATM indeed, did not change p-ATR quantity after treatment and upon 24 h of recovery. In contrast, WT hT significantly decreased p-ATR protein amount only 24 h post drug treatment. It has been reported the ratio p-ATR/WLN because the ratio p-ATR/ATR is influenced by ATR amount, that changed during treatment. Total ATR and the ratio p-ATR/ATR are reported in Fig. S5. Red asterisks refer to intra sample comparison, while the black ones refer to the statistical comparison with the control cell line UTD (Friedman test followed by Dunn’s test). Graphs show mean with SEM, *n* = 6

It is known that H2AX is also phosphorylated by ATR in response to replication stress [46]. Phosphorylated (Ser428) and total ATR were detected by WB analyses in all the tested cell lines with or without bleomycin treatment (Figs. 4 and S5b, respectively). p-ATR/ATR is reported in Fig. S5c, but it did not reflect the real content of these proteins in the tested cell lines because both total and phosphorylated ATR changed upon treatment.

In UTD, p-ATR/WLN ratio resulted constantly elevated, independently of bleomycin treatment (Fig. 4), while the ATR protein enhanced at 3 h post drug (Fig. S5b). Interestingly, all TD cells showed a reduced quantity of total p-ATR (Fig. 4) and a very low amount of ATR protein (Fig. S5b) than UTD at basal condition. After 3 h of bleomycin treatment, total amount of p-ATR increased only in TD 3-52 (Fig. 4), while the total amount of ATR increased in TD 3-52, TD 4-53, and TD SINT (Fig. S5b). After the 24 h recovery, TD 3-52, TD 4-53, and TD SINT showed higher amounts of p-ATR than their basal counterparts (Fig. 4). The same outcome was observed concerning total ATR (Fig. S5b). TD miniATM resulted practically uninvolved in ATR dynamics, since p-ATR and ATR proteins did not change after bleomycin treatment (Figs. 4 and S5).

On the contrary, WT hT cells displayed a statistically significant reduction of both p-ATR/WLN ratio (Fig. 4) and ATR/WLN ratio (Fig. S5b) after 24 h from the treatment.

### ATM variants activity on cell cycle targets

**Fig. 5:**
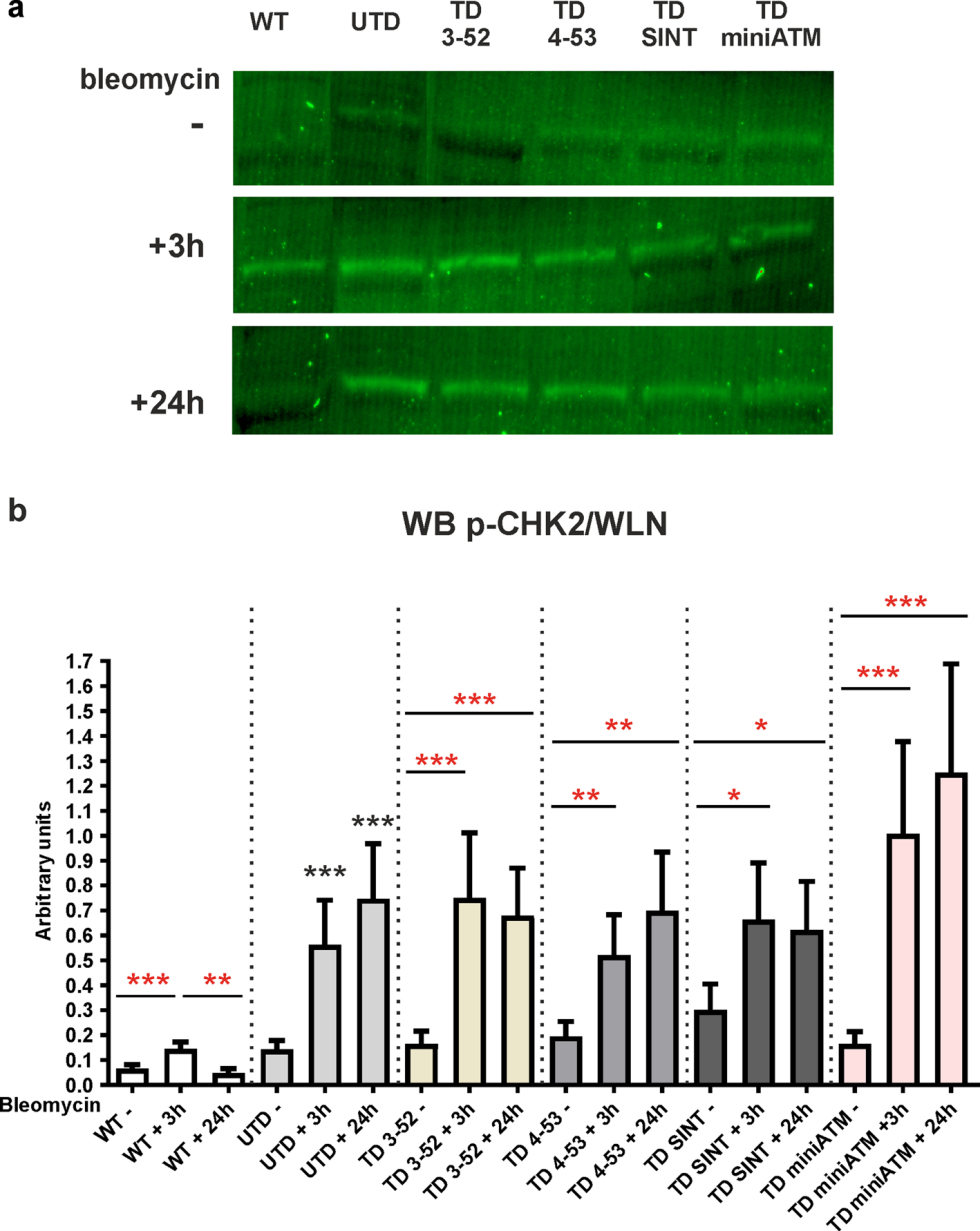
ATM targets CHK2 is activated by bleomycin in all AT 648 hT transduced cells. pCHK2 detection by WB. **a** Representative image and **b** quantification in all the tested samples, treated with bleomycin for 3 h and then kept in recovery course for 24 h. In WT hT cells, CHK2 phosphorylation was higher at 3 h of drug treatment compared to its basal counterpart, while it statistically decremented after the recovery condition. In all TD cells, there was an increase of CHK2 phosphorylation after 3 h bleomycin treatment, while in contrast to WT hT cells, the phosphorylation remained unaltered 24 h post treatment, indicating the non-fully activation of CHK2 in these cells. Only TD 3-52 and TD SINT decrease p-CHK2, but not in a statistically significant way. CHK2 phosphorylation has also been noticed in UTD both after 3 h of treatment and after 24 h of recovery condition, probably due to the ATR activity in these cells. This crosstalk has been verified in Fig. S6a and b, since CHK2 phosphorylation has been significantly reduced in UTD after the addition of VE-821. Red asterisks refer to intra sample comparison, while the black ones refer to statistical comparison with the control cell line UTD (Friedman test followed by Dunn’s test). Graphs show mean with SEM, *n* = 5

To further survey the biological functionality of the ATM variants, some other ATM downstream substrates involved in cell cycle checkpoint, were tested. The Checkpoint kinase 2 (CHK2) is activated by ATM in response to DNA DSBs [47, 48]. Accordingly, we examined the ability of ATM variants to activate this checkpoint kinase by analyzing the CHK2 phosphorylation at Thr68 after exposure to bleomycin by WB (Fig. 5). CHK2 phosphorylation is increased after 3 h of bleomycin treatment in all TD cells, while it seemed to decrease 24 h post drug only in TD 3-52 and TD SINT, with the same behavior of WT hT cells, despite not in a statistically significant way. Similarly, UTD presented p-CHK2, probably due to the crosstalk signaling between ATM/ATR pathways [49].

**Fig. 6:**
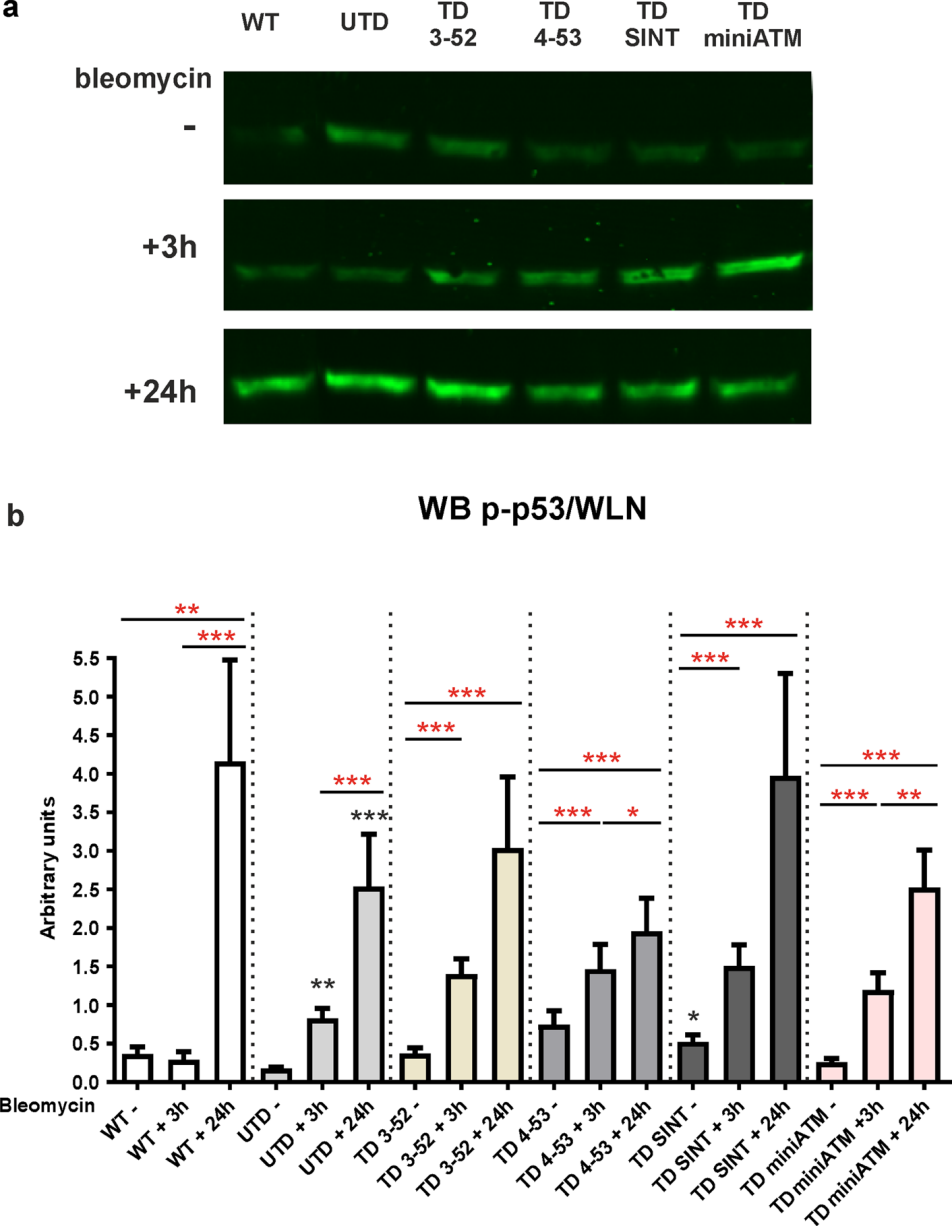
All ATM variants are able to phosphorylate p53, a further ATM downstream target after bleomycin stimulation. P-p53 detection by WB **a** representative image and **b** quantification in all tested samples. WT hT cells did not show p53 phosphorylation 3 h post bleomycin, while they statistically incremented p-p53 only after 24 h of recovery, since p53 activation is slower than CHK2. In all TD cells, p53 phosphorylation statistically incremented after 3 h of bleomycin compared to their untreated condition, while only TD 4-53 and TD miniATM statistically enhanced after the recovery condition in comparison to their treated counterparts. The improvement of p53 phosphorylation also in UTD, at 3 h of bleomycin treatment and after 24 h of recovery, is probably ascribed by ATR that could influence the assay. In fact, using VE-821 there was a decrease of p-p53 in UTD, as reported in fig S6a and c. Red asterisks refer to intra sample comparison, while the black ones refer to statistical comparison with the control cell line UTD (Friedman test followed by Dunn’s test). Graphs show mean with SEM, *n* = 5

An additional ATM downstream target is the tumor suppressor p53 [50], which once phosphorylated becomes stabilized and promotes its transcriptional activity. The phosphorylation of p53 by ATM at Ser15 was assayed by WB (Fig. 6), and the ratio p-p53/WLN was found to be enhanced after 3 h of bleomycin treatment in all TD cells, but not in WT hT cells, and showed its persistent activation 24 h post drug treatment in all the tested cell lines. However, p53 was found activated also in UTD. To evaluate CHK2 and p53 phosphorylation by ATM/ATR crosstalk, ATR inhibition by VE-821 was performed (Fig. S6) [28, 29]. UTD showed a strong CHK2 phosphorylation inhibition when cells were co-exposed with VE-821 and bleomycin. In contrast, CHK2 phosphorylation resulted unaltered in WT hT and in all TD cells. Concerning p53, there was a robust decrease of its phosphorylation in UTD, which was not observed in WT hT and TD cells. This suggested that p53 is not only induced by ATM after 3 h of bleomycin treatment in all tested cells.

### Autophagy is affected in AT 648 hT transduced cells

**Fig. 7:**
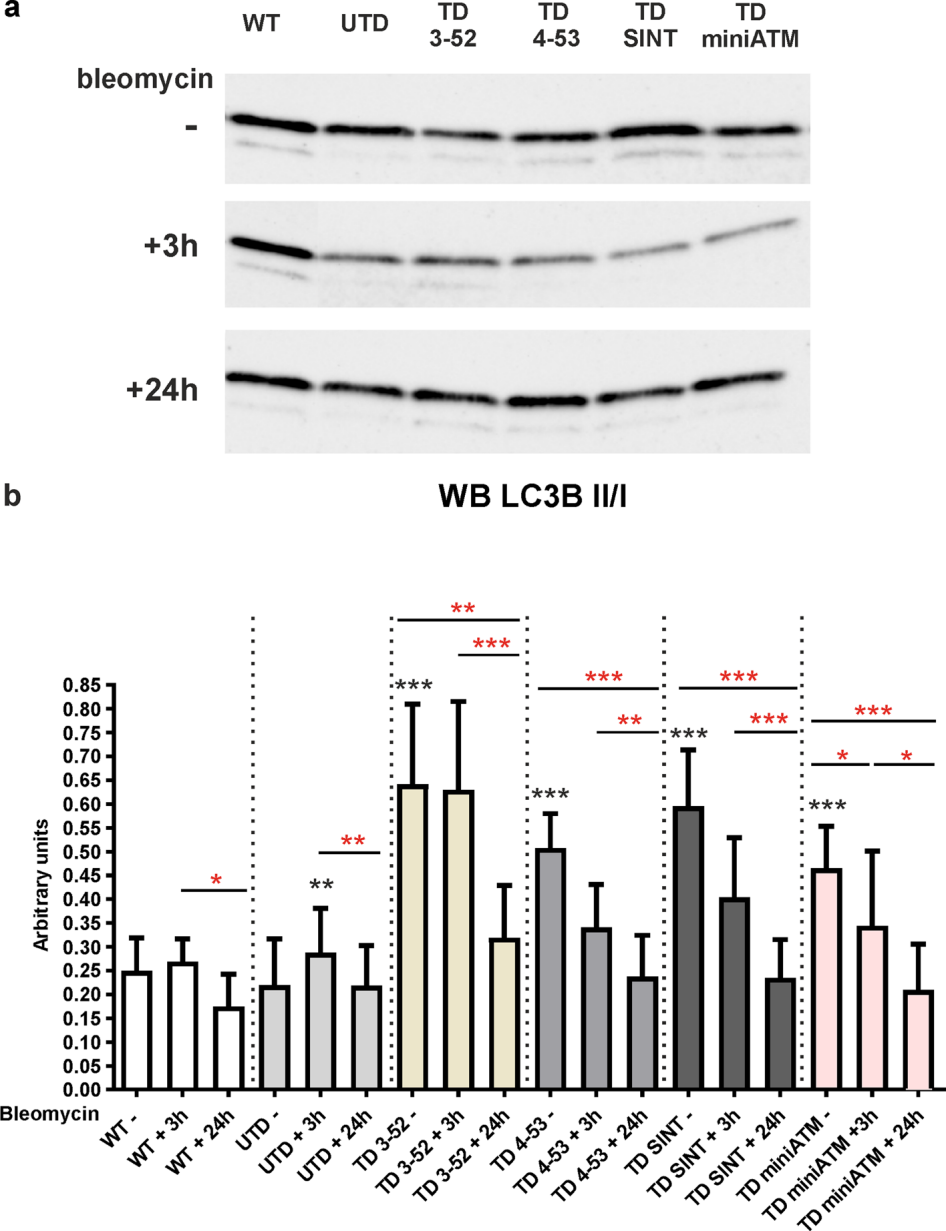
Autophagy marker LC3B is impacted by the presence of ATM variants in AT 648 hT cells. **a** LC3B-I and II typical WB image and **b** LC3B II/I quantification in all tested cells treated with bleomycin for 3 h and then kept in recovery course for 24 h. LC3B II/I ratio was not affected by bleomycin stimulation in WT hT cells, while it is reduced upon 24 h of recovery. All TD cells showed higher LC3B II/I ratio compared to UTD at basal conditions, suggesting that when ATM variants are present, LC3B II lipidation is promoted. When bleomycin is added, the ratio decreased in a statistical way only in TD miniATM, while it significant decreased after the recovery condition in all TD cells. UTD boosted LC3B II/I ratio at 3 h post drug treatment with a concomitant reduction of LC3B-I amount (LC3B I quantification is reported in Fig. S7), inferring the non-degradation of LC3B-II in these cells. The ratio LC3B II/I also decreased in UTD upon 24 h of recovery. Red asterisks refer to intra sample comparison, while the black ones show statistical comparison with the control cell line UTD (Friedman test followed by Dunn’s test). Graphs show mean with SEM, *n* = 7

It has recently been ascertained that ATM exhibits extra-nuclear functions, and since it is known that AT cells have an impairment in the autophagy process, particularly in the fusion between autophagosomes and lysosomes [51–53], we decided to investigate the autophagic flux in AT 648 hT transduced cells. Microtubule-associated protein light chain 3 (LC3) expression was used as an autophagy marker [54], performing a WB using antibody anti-LC3B (Fig. 7). LC3B I quantification is reported in Fig. S7.

In UTD at basal condition, the ratio LC3B II/I was lower than TD cells (Fig. 7), while LC3B I is increased compared to TD cells (Fig. S7). Under bleomycin treatment the ratio II/I increased (Fig. 7), whereas LC3B-I reduced its quantity (Fig. S7). Upon 24 h of recovery the LC3B II/I ratio decreased (Fig. 7), while LC3B-I enhanced its amount (Fig. S7).

When bleomycin was added to TD cells, the LC3B II/I ratio seems to be decreasing, but in a statistically significant manner only in TD miniATM (Fig. 7). Accordingly, a reduced quantity of LC3B-I was observed in the same sample (Fig. S7). After 24 h of recovery, the LC3B II/I ratio was statistically lower (Fig. 7), and concomitantly LC3B-I boosted its amount in all TD cells compared to their basal counterparts (Fig. S7). In contrast, in WT hT cells the ratio LC3B II/I decreased only at 24 h recovery condition (Fig. 7), while no changes of LC3B I was observed during the treatment (Fig. S7). TD 3-52 showed the most inconsistent LC3B behavior compared to WT hT, and to the other TD cells.

**Fig. 8:**
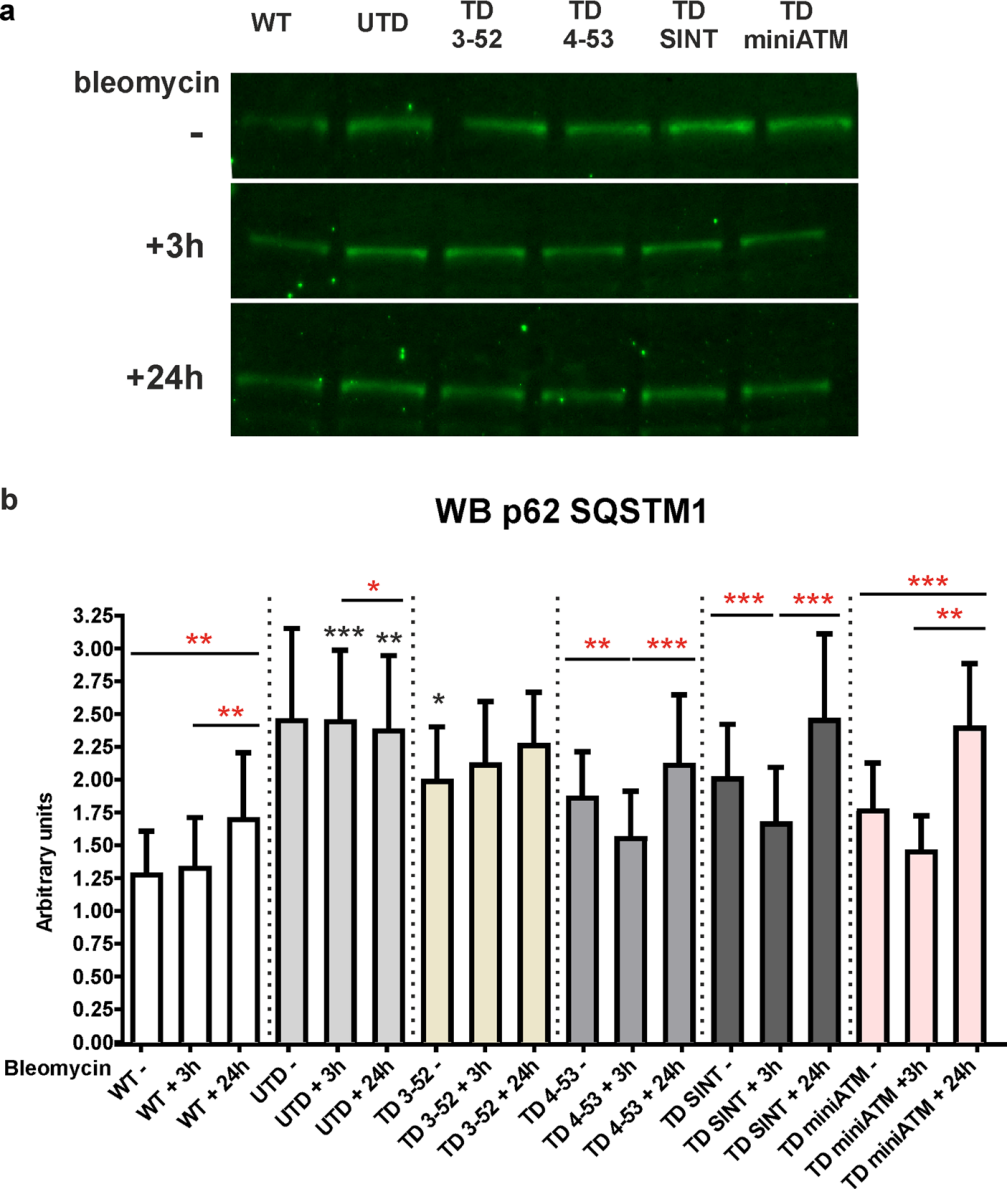
p62 analysis confirms autophagy impairment in UTD **a** p62-SQSTM1 representative images and **b** WB quantification in all the tested cell lines treated with bleomycin for 3 h and then kept in recovery course for 24 h. WT hT cells displayed the same quantity of p62 both at basal and after drug treatment but increased its amount upon recovery condition. TD cells presented a reduced amount of p62 in comparison with UTD at basal condition, despite being significant only compared to TD 3-52. After bleomycin treatment TD 4-53 and TD SINT degraded p62, improving the autophagy flux and supporting LC3B results. In contrast, p62 quantity is not affected by the treatment in TD 3-52 and TD miniATM. After 24 h of recovery, it enhanced in TD 4-53, TD SINT and TD miniATM. In UTD, p62 enhanced after 3 h of drug treatment and decreased after the recovery condition. Red asterisks refer to intra sample comparison, while the black ones show statistical comparison with the control cell line UTD (Friedman test followed by Dunn’s test). Graphs show mean with SEM, *n* = 7

Autophagy flux improvement by TD cells was also confirmed by the Sequestosome 1 (SQSTM1/p62) analysis, evaluated by WB (Fig. 8), the degradation of which reveals a progression of the autophagic process [55, 56]. Under basal conditions, p62 protein was elevated in UTD compared to TD 3-52 and to WT hT cells (Wilcoxon test for repeated measures). On the contrary, TD 4-53 and TD SINT presented a significant decrease in p62 content during 3 h of drug treatment, bringing its amount similar to those found in WT hT cells. Only TD 3-52 and TD miniATM did not statistically differ to the corresponding untreated cell line. The increment of p62 at 24 h was noticed in the TD 4-53, TD SINT and TD miniATM. In WT hT cells p62 quantity did not change after 3 h drug treatment and it increased after a 24-h recovery.

Autophagy also plays a key role in preserving the cellular proteostasis when endoplasmic reticulum (ER) stress occurs, leading to removal of damaged proteins [57, 58]. Calreticulin (CALR), a quality control chaperone, is induced under ER stress and could couple ER stress to autophagy, interacting with LC3B [59]. The amount of CALR was detected by WB assay in WT hT and AT 648 hT transduced and untransduced cells with or without bleomycin treatment (Fig. S8). At basal condition, UTD showed a CALR overexpression compared to TD cells, an indicator of ER stress in AT cells. When bleomycin is added, TD 3-52, TD 4-53 and TD SINT did not change their CALR amount after 3 h of drug treatment, WT hT cells demonstrated identical behavior. On the contrary, UTD and TD miniATM displayed a statistically significant decrease after 3 h of drug treatment. In agreement with other autophagy markers analyzed, we noticed an enhancement of CALR expression in all tested cell lines at 24 h of recovery.

### ATM 4-53 and miniATM improve the scavenger activity in counteracting ROS production

**Fig. 9:**
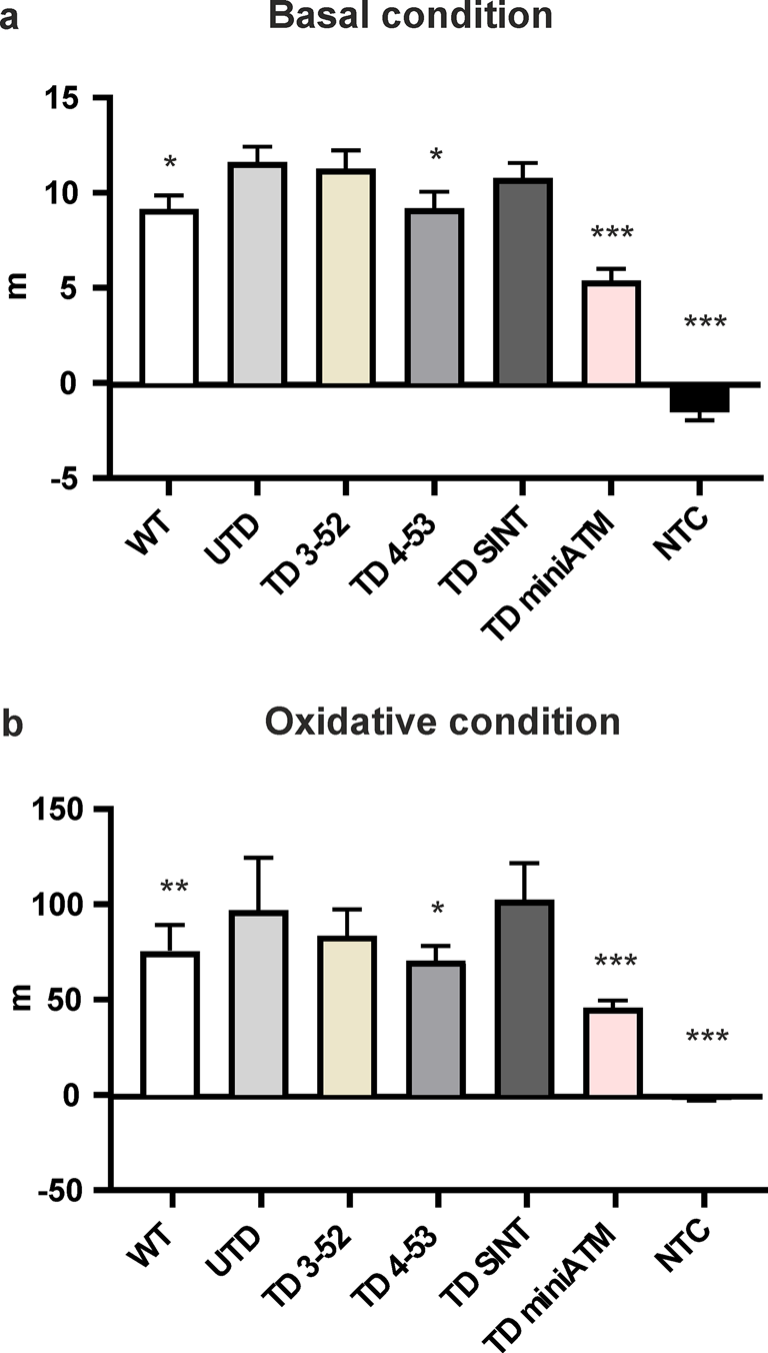
ATM 4-53 and miniATM counteracted ROS production. Slope quantification obtained from DCF oxidation kinetics of all the tested cell lines under **a** basal and **b** H_2_O_2_ stimulated conditions. WT hT cells presented a less oxidized cellular environment compared to UTD both under basal condition and under oxidative stimulus. Only TD 4-53 and TD miniATM showed less DCF oxidation kinetic than UTD under both conditions. In TD 3-52 and TD SINT, the amount of intracellular ROS did not statistically differ than UTD. Black asterisks refer to statistical comparison with the control cell line UTD (Kruskal–Wallis followed by Dunn’s test). Graphs show mean with SEM, *n* = 60

Guo et al. reported that ATM is also triggered by oxidative stress [6], independently of the DNA DSBs activation confirmed by its presence in the extra-nuclear compartments. Studies reported that AT phenotype is not only due to a defect in DNA-DSB response, but also due to a diminished control of ROS (reviewed by Ditch et al. and Watters et al. [7, 60]), in fact it was found that ATM-deficient cells are in a constant state of oxidative stress with higher levels of ROS [6, 61]. Based on this evidence, we aimed to investigate the antioxidant capacity in AT 648 hT transduced cells. Intracellular ROS levels were examined using the 2′-7′dichlorofluorescin diacetate (DCFH-DA) at basal condition (Fig. 9a) and with the addition of an oxidative stimulus (H_2_O_2_) (Fig. 9b), monitoring DCF oxidation kinetic. We confirmed the higher levels of ROS in UTD compared to WT hT cells under basal and treatment conditions. Interestingly, only TD 4-53 and TD miniATM showed a lower amount of intracellular ROS, indicating a greater antioxidant capacity in both basal and treated conditions. TD 3-52 and TD SINT did not show statistically significant differences in counteracting the presence of ROS.

### Mitochondria dysfunctions are restored by ATM variants

**Fig. 10:**
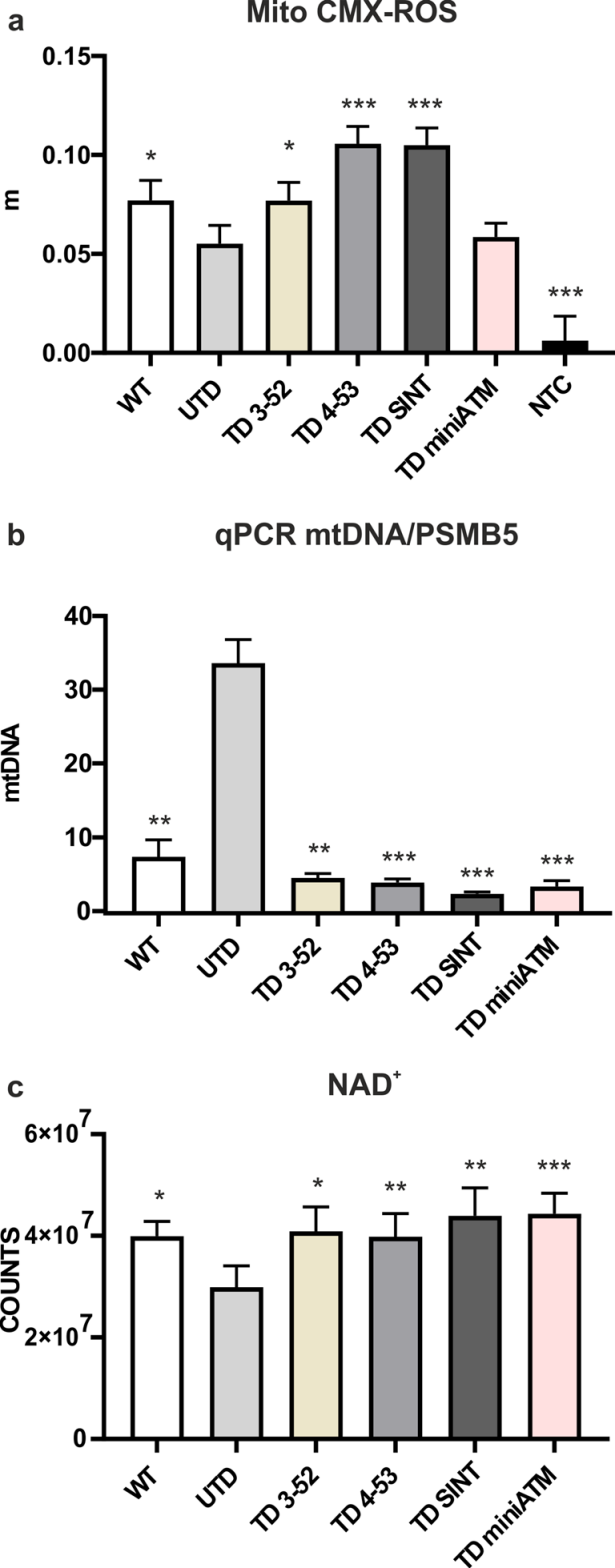
Restoration of mitochondrial functionality in AT 648 hT transduced cells. **a** Indirect membrane potential measures obtained by kinetic experiments in all the tested cell lines, stained with Mitotracker Red CMX-ROS. WT hT cells had a higher membrane potential than UTD and TD 3-52, TD 4-53 and TD SINT were able to recover it in a significant way. In contrast, TD miniATM did not present a significant increase in mitochondrial membrane potential compared to UTD. Black asterisks refer to statistical comparison with the control cell line UTD (Welch’s ANOVA test). Graphs show mean with SEM, *n* = 78. **b** Quantification of ND1/PSMB5 by quantitative PCR in all the tested cell lines. ND1, a specific mitochondrial gene, was amplified from the cytoplasmic fraction, while PSMB5 was used as a normalization from the nuclear fraction. WT hT cells and all TD cells presented a slight quantity of mtDNA in the cytoplasm compared to UTD, suggesting improved mitophagy when ATM variants are expressed in AT 648 hT cells. Black asterisks refer to statistical comparison with the control cell line UTD (Kruskal–Wallis test followed by Dunn’s test). Graphs show mean with SEM, *n* = 6. **c** NAD^+^ coenzyme was evaluated by mass spectrometry. The presence of ATM variants in AT 648 hT cells significantly incremented NAD^+^ quantity compared to UTD, raising it to the level found in WT hT cells. In contrast, UTD showed NAD^+^ depletion, confirming mitochondria dysfunctions. Black asterisks refer to statistical comparison with the control cell line UTD (Friedman test followed by Dunn’s test). Graphs show mean with SEM, *n* = 9

Mitochondria are the major source of ROS production through oxidative phosphorylation, and it has been reported that ATM is implicated in maintaining mitochondrial homeostasis [62, 63]. In fact, ATM protein is promptly triggered by mitochondrial dysfunctions, promoting mitophagy of aberrant and depolarized mitochondria [11, 64, 65]. Accordingly, the mitochondrial dysfunctions and the effects of ATM variants in AT 648 hT cells were tested. Mitochondrial membrane potential was indirectly evaluated by staining cells with MitoTracker Red CMX-ROS, whose entry depends on membrane potential, thus evaluating the dye entry kinetics (Fig. 10a), independently of mitochondria number. As expected, UTD showed a lower mitochondrial functionality compared to WT hT cells, in agreement with the high level of ROS found in these cells, while TD 3-52, TD 4-53 and TD SINT presented a faster dye entry, indicating a better mitochondria functionality than UTD. TD miniATM was not statistically significantly different than UTD.

Recently, Yang et al. reported that the classical senescence and inflammatory phenotype of AT cells might be triggered by the release of mtDNA in the cytoplasm from damaged mitochondria that are not properly removed [37]. Aberrant mitophagy has been ascribed to AT phenotype that in turn might be caused by NAD^+^ depletion [65]. Therefore, we first quantified the amount of mtDNA in the cytoplasmic fraction (Fig. 10b) and secondly, we quantified NAD^+^ coenzyme (Fig. 10c). Consistent with the study of Yang et al., we found an accumulation of mtDNA in the cytoplasm of UTD in comparison with WT hT cells. All TD cells statistically decremented the amount of mtDNA in the cytosol. Consistently, a lower amount of NAD^+^ was observed in UTD than in WT cells, while all TD cells were able to reinstate NAD^+^ levels.

### The expression of ATM variants in AT cells can alter HDAC4 localization

**Fig. 11:**
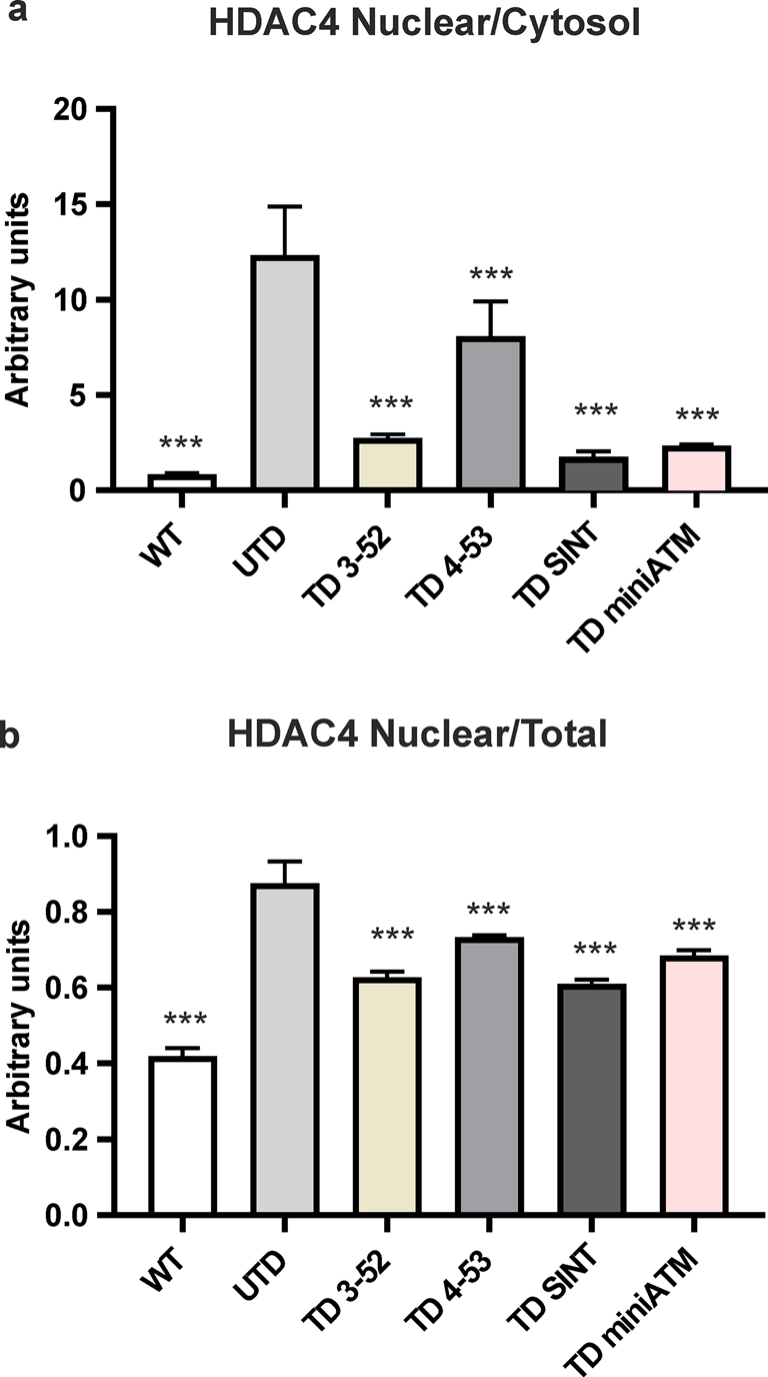
HDAC4 nuclear accumulation is decreased in TD cells. **a** Nuclear-cytosol and **b** nuclear/total HDAC4 shuttling in all the tested cell lines stained by IF. Since the HDAC4 nuclear/cytosol shuttle indirectly depends on ATM activity, this ability was evaluated. All TD cells were able to significantly alter its localization compared to UTD, lowering the amount of nuclear HDAC4 in terms of nuclear/cytosol and nuclear/total ratios to the level found in WT hT cells. Black asterisks refer to statistical comparison with the control cell line UTD. At least 200 nuclei from three independent experiments were counted for data processing (Kruskal–Wallis test followed by Dunn’s test). Graphs show mean with SEM

Previous studies concerning the HDAC4 dynamic in AT cells have shown that ATM deficiency leads to a nuclear HDAC4 accumulation, promoting neurodegeneration in AT neurons [66]. We decided to investigate the nucleus/cytosol HDAC4 shuttle in the stated cellular model. The nuclear accumulation was described by HDAC4 nuclear/cytosol and nuclear/total ratios. Performing indirect immunofluorescence (Figs. 11 and S9), we found an accumulation of HDAC4 in the nucleus in UTD compared to WT hT cells, as previously published [52]. All TD cells showed a reduced significant nuclear accumulation pattern, indicating they were able to modulate HDAC4 shuttle.

## Discussion

Ataxia Telangiectasia is a very severe disease with a pleiotropic phenotype, due to defects in DNA damage response and in extra-nuclear pathways, particularly in autophagy and mitophagy fluxes, both essential for neuronal survival. Neurodegeneration is the most critical problem of AT meaning that patients are often confined to a wheelchair by adolescence and have a very short life expectancy. Unfortunately, the mechanism leading to neurodegeneration is still not completely understood. Additionally, no treatment is available to cure these patients, with only supportive therapies to alleviate their multiple pains. In 2006, the serendipitous discovery of positive effects of glucocorticoid treatment on neurologic symptoms of AT patients [21] led to a new hope for a plausible treatment. The attempt to explain the mechanism behind dexamethasone positive effects in AT cells and patients is still ongoing, but the greatest outcome has been obtained by Menotta et al. [24]. They discovered that a reduced variant of the native ATM messenger ‘ATMdexa1’ is produced after drug stimulation through alternative splicing, that is translated into a shorter protein named ‘miniATM’ in only AT lymphoblastoid cells, retaining the kinase domain and therefore capable of partially rescuing the lack of ATM. The availability of blood samples provided by patients enrolled in the clinical trial [19] led them to the detection of ‘ATMdexa1’ also in vivo, and to the discovery of other ATM variants, containing additional domains to miniATM itself [25]. Prompted by the positive outcomes of miniATM, we proceeded with expressing ATM variants in a lentiviral system to assess whether they could overcome ATM absence in AT cells. We considered the two main roles of ATM: the response after DNA damage, to verify whether ATM variants were capable of propagating DNA signaling and ensuring DNA repair, and the extranuclear roles of oxidative stress protection, to evaluate ATM variants functions in the cytoplasm. DNA damage was induced through bleomycin treatment: 8 µg/mL of bleomycin was chosen, as the most effective concentration, corresponding to 3.2 Gy. Accordingly, several ATM targets were evaluated upon 3 h of continuous treatment, comparing TD cells to UTD, and using WT cells as a reference control. Moreover, a recovery condition of 24 h post bleomycin stimulation has been carried out to observe the capacity of ATM variants to recover DNA damage lesions. H2AX is phosphorylated by ATM, and it is considered the main sensor of DSBs, forming nuclear foci at the break sites [40, 43, 67]. Its phosphorylation followed by its rapidly dephosphorylation is a marker of DNA damage response (DDR) function, indicating a good propagation of damage signaling, avoiding permanent lesions [68]. Both phosphorylation and the analysis of foci obtained by immunofluorescence analysis were therefore investigated, considering that foci count is a more appropriate method to evaluate the regression of DNA damage. The enhanced phosphorylation of H2AX with an increased type I staining pattern after 3 h of drug in all TD cells led us to assume that ATM variants are activated under DNA breaks. Additionally, a decrease of its phosphorylation 24 h post treatment with an increase type III staining pattern was noticed in all TD cells, even though this intensity reduction (Fig. 2) is not consistent with a reduction in foci number (Fig. 3b) in TD 3-52 and TD 4-53. On the contrary, TD SINT presented a reduced number of foci (Fig. 3b) consistent with its intensity reduction 24 h post drug (Fig. 2), indicating correction of the lesions, in agreement with WT hT behavior. Similarly, TD miniATM presented a significance reduction of foci number 24 h post treatment, but with a lower fluorescence intensity than TD SINT after 3 h treatment (Fig. 2). This could be related, as reported in previous studies [68–70], to a mix signal where lesions are induced and repaired at the same time upon continuous treatment with bleomycin. However, the elevated persistence of foci number 3 h post drug in TD miniATM (Fig. 3b), led us to exclude this hypothesis and to propose that the observed foci number reduction after 24 h of recovery might be due to its decreased capability in activating H2AX. TD 3-52 presented a higher percentage of type I staining pattern 24 h post drug compared to other TD cells, since we observed a pan-nuclear staining of foci also in the recovery condition without a reduced number of foci. TD 4-53, on the contrary, decreased the type I and II pattern after the 24 h recovery course but, despite a lower fluorescence intensity, TD 4-53 did not show a statistically diminution of foci amount. The aa from 91 to 97 in the TAN domain in the N-terminal of ATM protein are essential for its interaction with p53, LKB1 and BRCA1 proteins [71] to propagate the DNA damage signaling, and this could account for the slower repair efficiency which occurred in TD 3-52, since it does not possess these aa. The ATM 4-53 variant instead contains these essential aa, but it does not contain the leucin zipper motif [72, 73], which is also required for ATM binding with interacting proteins and for targeting ATM to the proper cellular localization [74], that could explain its delay in the DDR, even if more performing than ATM 3-52 variant upon DNA damage. These domains are both present in the ATM SINT variant designed in silico*.* In addition, the native active monomer presents the exposed N-terminal domain to be able to interact with its substrates. In this way, it is in proximity of the C-terminal kinase domain, promoting the phosphorylation of its target [75]. This space orientation likely occurs also in ATM SINT and ATM 4-53, explaining their improved functions in the DNA damage propagation and repair, even though this is to a lesser extent in ATM 4-53. In addition, the ATM variants reported here did not possess Ser1981 in the FRAP-ATM-TRRAP (FAT) domain, responsible for the classical ATM activation pathway, therefore we can speculate that their mechanism of action might derive from the contribution of several cell components and maybe by the interaction with the endogenous native ATM protein, where the latter could be used as a scaffold for anchoring targets, implementing the function of the ATM variants.

Other considerations need to be taken for γH2AX behavior in UTD. We observed the same pattern of TD cells in increasing and decreasing H2AX phosphorylation after 3 h of bleomycin and 24 h, respectively. However, in UTD, the critical foci analysis always revealed a high number of type III foci at all time points, suggesting a different phosphorylation pattern. The absence of ATM led to a replication stress condition, that is responsible for ATR induction [46, 76] and subsequent H2AX phosphorylation in the UTD, independently of DSBs signaling, but dependent on replication blocks. Consequently, the foci types are only affected by the replication stress condition due to ATM absence, and not by bleomycin treatment. The constant presence of p-ATR in UTD justifies this hypothesis and influences the phosphorylation of H2AX, maintaining high fluorescence intensity but without reduced foci amount, indicating the persistence of unrepaired DNA lesions, that usually lead to cell death.

Desai et al*.* highlighted a downregulation in the ubiquitin proteasome system in AT cells [77], and since ATR is degraded by this system [53], it could be possible that this is the reason for its larger amount in UTD. In WT hT cells, the expression of p-ATR and ATR did not change after drug stimulation, probably because bleomycin treatment responds to ATM and not to ATR. In contrast, a same amount of p-ATR before and after the treatment is observed when ATM variants are expressed in AT cells (except for TD 3-52). This rules-out the possibility that H2AX may be phosphorylated by ATR in these cells, since p-ATR did not enhance consistently with the increased quantity of ATR after a drug stimulus and highlights the potential function of ATM variants to substitute the ATM response to DNA damage. Unexpectedly, TD miniATM did not behave like TD 3-52, TD 4-53 and TD SINT regarding ATR phosphorylation. We did notice a higher ATR content with respect to other TD cells at basal condition, but total and phosphorylated content of ATR kept stable, without being affected by bleomycin treatment. Similar amounts of ATR and p-ATR before and after the treatment in TD miniATM might reveal that miniATM variant could still require ATR after receiving a drug stimulus, indicating the persistence of replication stress which is moderated by the ATR protein.

Further evidence for ATM variants function in DDR were obtained by analyzing other ATM direct downstream targets, including CHK2 and p53 phosphorylation at Thr68 and Ser15, respectively. CHK2 Thr68 phosphorylation depends on ATM activity [78], while it is not indispensable for continuing its kinase activity [79, 80]. Accordingly, all TD cells were able to trigger Thr68 phosphorylation after treatment, but the persistence of its phosphorylation 24 h post bleomycin exposure might demonstrate the incapacity of TD cells to fully activate CHK2. Only TD 3-52 and TD SINT showed a hint of the same WT cell behavior, indicating a similarity to the pattern that occurs in WT cells after DNA damage. Additionally, ATM phosphorylation on p53 at Ser15 [50] was considered in this study. This is the best documented phosphorylation site, and it is the one responsible for inhibiting the interaction with its negative regulator murine double minute 2 (MDM2) [81]. In the reported experiments there is an increase of p53 phosphorylation in all TD cells and in WT hT cells upon 24 h of the treatment. This slow p53 activation was noted in other cell systems: human normal and AT (GM00363 and GM00648) fibroblast cell lines [82] and human colon carcinoma cells [83], where p53 phosphorylation persisted until 24–48 h of bleomycin treatment. This time-extended phosphorylation was also found in clear cell renal carcinoma cells treated with etoposide for 30 min and kept in recovery course for 24 h [84]. It is known that ATM acts on p53 not only in a direct manner but also indirectly, phosphorylating other upstream p53 proteins that contribute to its stability and function [8, 85]. Consequently, its phosphorylation may occur more slowly than CHK2, since it does not depend merely on ATM, and it is still required for p21 transcription [86], displaying its role in both the establishment and support of cell cycle arrest [87, 88]. Concerning the cell cycle checkpoint proteins in UTD, we still noticed the crosstalk between ATM/ATR, that affected the assay and led to the misinterpretation of the results. As previously reported, p53 phosphorylation at Ser15 is induced by ATR in AT cells when DNA damage occurred [89] and under hyperoxia in AT primary fibroblast cells [90]. Carranza et al. also observed a slight activation of p53 in AT fibroblast cells after irradiation [91]. Moreover, increased ATR activity was observed in the absence of ATM, being responsible for CHK2 phosphorylation at Thr68 in response to ionizing radiation in vitro [49]. Interestingly, it has been described that ATM-deficient cells displayed activation of common ATM dependent targets after DSBs, albeit to a lesser extent. This assumes an enhanced action of another member of the trinity ATM, ATR and DNA-dependent protein kinase catalytic subunit (DNA-PK) when one is missing [92]. These outcomes led us to propose that also in our cellular model p-CHK2 and p-p53 might be induced by ATR in UTD [93]. This mechanism has been confirmed by using the ATR kinase inhibitor VE-821 [28, 29]. In the absence of ATR activity, CHK2 reduced its phosphorylation in UTD confirming the crosstalk between ATM/ATR, while in WT hT and in all TD cells it remained phosphorylated, highlighting the ability of ATM variants to activate the cell cycle checkpoint CHK2. In contrast, ATR activity on p53 phosphorylation is robust after 3 h, suggesting a slower activation of p53 by ATM. Nevertheless, WT hT and TD cells behaved in the same way, while p53 was almost completely unphosphorylated in UTD. These data demonstrated that the crosstalk between ATM/ATR exists, but they confirm the role of ATM variants in phosphorylating these targets in TD cells.

Another essential mechanism that is impaired in AT cells is the autophagy flux, since autolysosomes are not appropriately formed and degraded, hence inhibiting the removal of misfolded proteins and damaged organelles [51, 52]. The results reported here, concerning LC3B and p62, confirmed the basal accumulation of autophagosomes in UTD, that are unable to fuse to lysosomes, displaying a block in the autophagy flux even after an autophagy stimulus. LC3B-II is responsible for the movement of autophagosomes towards lysosomes [94]: TD cells appeared to promote the conversion of LC3B-I to the lipidated form LC3B-II at basal condition and further after drug stimulus, helping autophagosome-lysosomes trafficking. It is noted that LC3B amount should also be evaluated by blocking experiments, but nonetheless the analysis of p62 degradation in TD 4-53 and TD SINT suggests that a proper vesicle fusion occurred. Further evidence of autophagy impairment in UTD was obtained by observing an increased ER stress as measured by CALR overexpression [59] indicating a condition of proteostatic stress, in agreement with previous papers that demonstrated alteration of protein homeostasis in the absence of ATM [13, 95]. This condition may be restored by expressing ATM variants in AT cells, that presented a smaller amount of CALR, at least at basal conditions. In contrast, WT hT cells appeared to be unaffected by bleomycin for LC3B, p62 and CALR autophagy targets. This is probably since native ATM can counteract the DNA break sites induced by the treatment, before influencing the autophagy pathway. All these results demonstrated an effort of TD cells to mimic WT hT cell pattern, partially rescuing the autophagy impairment.

A recent discovery pinpointed ATM activation by oxidative stress, in an independent manner from its activation by DNA damage, thus contributing to redox homeostasis regulation [6]. Therefore, any impairment in this mechanism leads to oxidative stress condition, with deleterious results especially in post-mitotic neurons. By investigating the antioxidant capacity, we were able to observe, as expected, high levels of ROS in the UTD, that decreased only in the presence of ATM 4-53 and miniATM. Interestingly, looking more specifically at the main ROS production organelle, we did observe a decreased mitochondrial membrane potential with a greater release of mtDNA in the cytoplasm and also a NAD^+^ deficiency in UTD, indicating mitochondria dysfunctions. Recent studies correlated ATM localization to mitochondria in normal human fibroblasts and demonstrated its activation after mitochondrial dysfunctions, suggesting a direct action of ATM on mitochondrial proteins. ATM absence, indeed, led to increased mitochondrial ROS, reduced mitochondrial functions, and decreased mitophagy, leading to the conclusion that AT disease should be considered also as a mitochondrial disease [11, 62, 63]. In contrast, the increased mitochondrial membrane potential in TD cells indicates the restoration of mitochondrial dysfunctions in AT cells. Unexpectedly, TD miniATM showed the lowest levels of ROS and the worst mitochondria membrane potential suggesting it has a more general role in counteracting oxidative stress, rather than only being localized in the mitochondria. However, consistent with the positive effects obtained with ATM variants in enhancing mitochondria membrane potential, we also found less mtDNA accumulation in the cytoplasm of all TD cells, including miniATM, that revealed the absence of aberrant mitochondria due to improved mitophagy. Additionally, NAD^+^ amounts was also increased in all TD cells, contributing to improved mitophagy flux, as shown in previous works in AT fibroblasts and AT neurons [37, 65]. Neurons contain numerous mitochondria, to sustain their energy demand and function, therefore any aberrant mitochondrion that is not properly removed might affect neuron energy production and neuronal survival. Furthermore, the crosstalk between mitochondrial dysfunctions and DNA damage has been highlighted by Fang et al., demonstrating that NAD^+^ supplementation is critical for both restoring mitochondrial function and mitophagy flux and reversing DNA lesions in AT models [65]. Similarly, the UTD displayed persistent DNA damage with lower mitochondrial functionality and NAD^+^ deficiency; all these features were restored by ATM variants expressed in AT cells that displayed higher NAD^+^ levels, like those observed in WT hT cells.

We cannot exclude the likely activation of the proposed ATM variants following oxidative stress condition as a dimer formed by oxidation of two monomers of cysteines at position 2991 in the FATC domain, since all ATM variants possess the full FATC domain. This last event might explain the beneficial role of the ATM variants in stabilizing mitochondrion activity and therefore restoring redox homeostasis. ATM 4-53 appeared to be the best ATM variant in counteracting cytoplasmic oxidative stress and mitochondrial dysfunctions, while the other ATM variants seemed to have a more specific action localized in mitochondrion. However, these cytoplasmic restored functions by ATM variants could contribute by supporting the antioxidant capacity of neurons and preventing cerebella degeneration.

Neurodegeneration has also been linked to HDAC4 nuclear accumulation in the absence of ATM [66], however, we were able to demonstrate that dexamethasone could act on HDAC4 nuclear accumulation in a different manner, leading to an unexpected outcome of autophagy improvement in AT fibroblast cells [52]. TD cells had the ability to reverse HDAC4 localization, as we observed less HDAC4 amount in the nucleus than UTD. It is likely that ATM variants, acting as native ATM in healthy cells, could phosphorylate and inactivate protein phosphatase 2A (PP2A) [66, 96], maintaining HDAC4 phosphorylation and its cytoplasmic distribution [97], even though they were not able to achieve WT levels.

In conclusion, Table 1 summarizes the functions of ATM variants when expressed in AT cells. It is possible to observe that TD 4-53 and TD SINT gave the highest values. TD 4-53 performed better in regard to cytoplasmic functions but TD SINT, was more efficient for foci reduction after DNA damage.

**Table 1:** A summary table with all the tested activities of TD cells. ATM 4-53 and ATM SINT variants are an optimal starting point for further applications in the treatment of AT patients

	γH2AX	p-CHK2	p-p53	Autophagy recovery	Oxidative stress recovery	Mitochondria recovery	HDAC4 shuttle
TD 3-52	+−	+	+	+−	−	+	+
TD 4-53	+−	+−	+	+	+	+	+
TD SINT	++	+	+	+	−	+	+
TD miniATM	+−	+−	+	+	+	+−	+

Together, these data support the beneficial roles of ATM variants in restoring cellular functionalities missing in AT patients. Furthermore, the smaller size of ATM variants cDNA overcomes the cargo limit of approved gene therapy vectors, improving the efficiency of viral particles production and infection efficiency compared with the whole ATM gene, as reported by Carranza et al., who constructed a lentiviral vector containing a full-length ATM capable of rescuing AT deficiencies, but with a low transduction efficiency [91]. Hence, ATM variants could be used in gene therapy, or their administration could also be achieved by using nanoparticle delivery or vesicle delivery, both of which are less immunogenic, less expensive, and more efficient in crossing the blood–brain barrier. To date, there are no reports of nanoparticle application for the treatment of AT.

Further studies should be performed to fully understand their functions, but this work is an optimal proof of concept for considering ATM variants in potential treatments for AT patients, providing important data to help reverse this devastating disease.

## Supplementary Information

Below is the link to the electronic supplementary material.

Supplementary file1 (PDF 1678 KB)

## Data Availability

All data generated or analyzed during this study are included in this published article (and its Supplementary Information files) and available from the corresponding author on reasonable request. Please note that part of the results is patent pending.

## Abbreviations

AT: Ataxia telangiectasia
*ATM*: Ataxia telangiectasia mutated
ATR: Ataxia telangiectasia and Rad3 related
CALR: Calreticulin
CHK2: Checkpoint kinase 2
DDR: DNA damage response
Dex: Dexamethasone
DNA-PK: DNA-dependent protein kinase catalytic subunit
DSBs: DNA double-strand breaks
ER: Endoplasmic reticulum
EryDex system: Dexamethasone sodium phosphate delivered through autologous red blood cells
FAT: FRAP-ATM-TRRAP
FAT-C: FAT-C-terminal
HDAC4: Histone deacetylase 4
H2AX: H2A histone family, member X
γH2AX: Phosphorylated form of H2AX
LC3: Microtubule-associated protein light chain 3
MRN: Meiotic recombination protein-11 (Mre11)/Rad50/Nijmegen Breakage Syndrome-1 (Nbs1)
mTOR: Mammalian target of rapamycin
PIKK: Phosphatidyl inositol 3-kinase-related kinases
PI3K: Phosphatidyl inositol 3-kinase
ROS: Reactive oxygen species
SQSTM1/p62: Sequestosome 1
TAN: Telomere-length maintenance and DNA damage repair
TD: AT 648 hT transduced cells
TD 3-52: AT 648 hT cell lines transduced with ATM 3-52
TD 4-53: AT 648 hT cell lines transduced with ATM 4-53
TD SINT: AT 648 hT cell lines transduced with ATM SINT
TD miniATM: AT 648 hT cell lines transduced with miniATM
UTD: AT 648 hT untransduced cells
