# Supplementary material for: New human ATM variants are able to regain ATM functions in ataxia telangiectasia disease
Source: Cell Mol Life Sci. 2022 Nov 23;79(12):601. doi: 10.1007/s00018-022-04625-3 (PMC9691487; doi:10.1007/s00018-022-04625-3)
Supplement: Supplementary file 1 — Supplementary file1 (PDF 1678 KB) [file 18_2022_4625_MOESM1_ESM.pdf]

New human ATM variants are able to regain ATM functions in Ataxia Telangiectasia disease

Cellular and Molecular Life Sciences

Anastasia Ricci, Federica Biancucci, Gianluca Morganti, Mauro Magnani and Michele Menotta

Department of Biomolecular Sciences, University of Urbino "Carlo Bo", Via Saffi 2, 61029, Urbino, Italy,

Corresponding Author: Anastasia Ricci, Department of Biomolecular Sciences, University of Urbino "Carlo Bo", Via Saffi 2, 61029 Urbino, Italy. Email: anastasia.ricci@uniurb.it.

Anastasia Ricci ORCID 0000-0001-6333-5797

Mauro Magnani ORCID 0000-0001-6456-6626

Michele Menotta ORCID 0000-0001-5206-6296

Supplementary Materials

ATM 3-52:

```
atga gtc tag tac tta atg atc tgc tta tct gct gcc gtc aac tag aac atg ata gag cta
  -   V   -   Y   L   M   I   C   L   S   A   A   V   N   -   N   M   I   E   L
cag aac gaa aga aag aag ttg aga aat tta agc gcc tga ttc gag atc ctg aaa caa tta
 Q   N   E   R   K   K   L   R   N   L   S   A   -   F   E   I   L   K   Q   L
aac atc tag atc ggc att cag att cca aac aag gaa aat att tga att ggg atg ctg ttt
 N   I   -   I   G   I   Q   I   P   N   K   E   N   I   -   I   G   M   L   F
tta gat ttt tac aga aat ata ttc aga aag aaa cag aat gtc tga gaa tag caa aac caa
 L   D   F   Y   R   N   I   F   R   K   K   Q   N   V   -   E   -   Q   N   Q
atg tat cag cct caa cac aag cct cca ggc aga aaa aga tgc agg aaa tca gta gtt tgg
 M   Y   Q   P   Q   H   K   P   P   G   R   K   R   C   R   K   S   V   V   W
tca aat act tca tca aat gtg caa aca gaa gat cga aca gag gct gca aat aga ata ata
 S   N   T   S   S   N   V   Q   T   E   D   R   T   E   A   A   N   R   I   I
tgt act atc aga agt agg aga cct cag atg gtc aga agt gtt gag gca ctt tgt gat gct
 C   T   I   R   S   R   R   P   Q   M   V   R   S   V   E   A   L   C   D   A
tat att ata tta gca aac tta gat gcc act cag tgg aag act cag aga aaa ggc ata aat
 Y   I   I   L   A   N   L   D   A   T   Q   W   K   T   Q   R   K   G   I   N
att cca gca gac cag cca att act aaa ctt aag aat tta gaa gat gtt gtt gtc cct act
 I   P   A   D   Q   P   I   T   K   L   K   N   L   E   D   V   V   V   P   T
atg gaa att aag gtg gac cac aca gga gaa tat gga aat ctg gtg act ata cag tca ttt
 M   E   I   K   V   D   H   T   G   E   Y   G   N   L   V   T   I   Q   S   F
aaa gca gaa ttt cgc tta gca gga ggt gta aat tta cca aaa ata ata gat tgt gta ggt
 K   A   E   F   R   L   A   G   G   V   N   L   P   K   I   I   D   C   V   G
tcc gat ggc aag gag agg aga cag ctt gtt aag ggc cgt gat gac ctg aga caa gat gct
 S   D   G   K   E   R   R   Q   L   V   K   G   R   D   D   L   R   Q   D   A
gtc atg caa cag gtc ttc cag atg tgt aat aca tta ctg cag aga aac acg gaa act agg
 V   M   Q   Q   V   F   Q   M   C   N   T   L   L   Q   R   N   T   E   T   R
aag agg aaa tta act atc tgt act tat aag gtg gtt ccc ctc tct cag cga agt ggt gtt
 K   R   K   L   T   I   C   T   Y   K   V   V   P   L   S   Q   R   S   G   V
ctt gaa tgg tgc aca gga act gtc ccc att ggt gaa ttt ctt gtt aac aat gaa gat ggt
 L   E   W   C   T   G   T   V   P   I   G   E   F   L   V   N   N   E   D   G
gct cat aaa aga tac agg cca aat gat ttc agt gcc ttt cag tgc caa aag aaa atg atg
 A   H   K   R   Y   R   P   N   D   F   S   A   F   Q   C   Q   K   K   M   M
gag gtg caa aaa aag tct ttt gaa gag aaa tat gaa gtc ttc atg gat gtt tgc caa aat
 E   V   Q   K   K   S   F   E   E   K   Y   E   V   F   M   D   V   C   Q   N
ttt caa cca gtt ttc cgt tac ttc tgc atg gaa aaa ttc ttg gat cca gct att tgg ttt
 F   Q   P   V   F   R   Y   F   C   M   E   K   F   L   D   P   A   I   W   F
gag aag cga ttg gct tat acg cgc agt gta gct act tct tct att gtt ggt tac ata ctt
 E   K   R   L   A   Y   T   R   S   V   A   T   S   S   I   V   G   Y   I   L
gga ctt ggt gat aga cat gta cag aat atc ttg ata aat gag cag tca gca gaa ctt gta
 G   L   G   D   R   H   V   Q   N   I   L   I   N   E   Q   S   A   E   L   V
cat ata gat cta ggt gtt gct ttt gaa cag ggc aaa atc ctt cct act cct gag aca gtt
 H   I   D   L   G   V   A   F   E   Q   G   K   I   L   P   T   P   E   T   V
cct ttt aga ctc acc aga gat att gtg gat ggc atg ggc att acg ggt gtt gaa ggt gtc
 P   F   R   L   T   R   D   I   V   D   G   M   G   I   T   G   V   E   G   V
ttc aga aga tgc tgt gag aaa acc atg gaa gtg atg aga aac tct cag gaa act ctg tta
 F   R   R   C   C   E   K   T   M   E   V   M   R   N   S   Q   E   T   L   L
acc att gta gag gtc ctt cta tat gat cca ctc ttt gac tgg acc atg aat cct ttg aaa
 T   I   V   E   V   L   L   Y   D   P   L   F   D   W   T   M   N   P   L   K
gct ttg tat tta cag cag agg ccg gaa gat gaa act gag ctt cac cct act ctg aat gca
 A   L   Y   L   Q   Q   R   P   E   D   E   T   E   L   H   P   T   L   N   A
gat gac caa gaa tgc aaa cga aat ctc agt gat att gac cag agt ttc aac aaa gta gct
```

```
 D   D   Q   E   C   K   R   N   L   S   D   I   D   Q   S   F   N   K   V   A
gaa cgt gtc tta atg aga cta caa gag aaa ctg aaa gga gtg gaa gaa ggc act gtg ctc
 E   R   V   L   M   R   L   Q   E   K   L   K   G   V   E   E   G   T   V   L
agt gtt ggt gga caa gtg aat ttg ctc ata cag cag gcc ata gac ccc aaa aat ctc agc
 S   V   G   G   Q   V   N   L   L   I   Q   Q   A   I   D   P   K   N   L   S
cga ctt ttc cca gga tgg aaa gct tgg gtg tga
 R   L   F   P   G   W   K   A   W   V   -
```

ATM 4-53:

```
atg agt cta gta ctt aat gat ctg ctt atc tgc tgc cgt caa cta gaa cat gat aga gct
 M   S   L   V   L   N   D   L   L   I   C   C   R   Q   L   E   H   D   R   A
aca gaa cga aag aaa gaa gtt gag aaa ttt aag cgc ctg att cga gat cct gaa aca att
 T   E   R   K   K   E   V   E   K   F   K   R   L   I   R   D   P   E   T   I
aaa cat cta gat cgg cat tca gat tcc aaa caa gga aaa tat ttg aat tgg gat gct gtt
 K   H   L   D   R   H   S   D   S   K   Q   G   K   Y   L   N   W   D   A   V
ttt aga ttt tta cag aaa tat att cag aaa gaa aca gaa tgt ctg aga ata gca aaa cca
 F   R   F   L   Q   K   Y   I   Q   K   E   T   E   C   L   R   I   A   K   P
aat gta tca gcc tca aca caa gcc tcc agg cag aaa aag atg cag gaa atc agt agt ttg
 N   V   S   A   S   T   Q   A   S   R   Q   K   K   M   Q   E   I   S   S   L
gtc aaa tac ttc atc aaa tgt gca aac aga aga gca cct agg cta aaa tgt caa gaa ctc
 V   K   Y   F   I   K   C   A   N   R   R   A   P   R   L   K   C   Q   E   L
tta aat tat atc atg gat aca gtg aaa gat tca tct aat ggt gct att tac gga gct gat
 L   N   Y   I   M   D   T   V   K   D   S   S   N   G   A   I   Y   G   A   D
tgt agc aac ata cta ctc aaa gac att ctt tct gtg aga aaa tac tgg tgt gaa ata tct
 C   S   N   I   L   L   K   D   I   L   S   V   R   K   Y   W   C   E   I   S
cag caa cag tgg tta gaa ggc ata aat att cca gca gac cag cca att act aaa ctt aag
 Q   Q   Q   W   L   E   G   I   N   I   P   A   D   Q   P   I   T   K   L   K
aat tta gaa gat gtt gtt gtc cct act atg gaa att aag gtg gac cac aca gga gaa tat
 N   L   E   D   V   V   V   P   T   M   E   I   K   V   D   H   T   G   E   Y
gga aat ctg gtg act ata cag tca ttt aaa gca gaa ttt cgc tta gca gga ggt gta aat
 G   N   L   V   T   I   Q   S   F   K   A   E   F   R   L   A   G   G   V   N
tta cca aaa ata ata gat tgt gta ggt tcc gat ggc aag gag agg aga cag ctt gtt aag
 L   P   K   I   I   D   C   V   G   S   D   G   K   E   R   R   Q   L   V   K
ggc cgt gat gac ctg aga caa gat gct gtc atg caa cag gtc ttc cag atg tgt aat aca
 G   R   D   D   L   R   Q   D   A   V   M   Q   Q   V   F   Q   M   C   N   T
tta ctg cag aga aac acg gaa act agg aag agg aaa tta act atc tgt act tat aag gtg
 L   L   Q   R   N   T   E   T   R   K   R   K   L   T   I   C   T   Y   K   V
gtt ccc ctc tct cag cga agt ggt gtt ctt gaa tgg tgc aca gga act gtc ccc att ggt
 V   P   L   S   Q   R   S   G   V   L   E   W   C   T   G   T   V   P   I   G
gaa ttt ctt gtt aac aat gaa gat ggt gct cat aaa aga tac agg cca aat gat ttc agt
 E   F   L   V   N   N   E   D   G   A   H   K   R   Y   R   P   N   D   F   S
gcc ttt cag tgc caa aag aaa atg atg gag gtg caa aaa aag tct ttt gaa gag aaa tat
 A   F   Q   C   Q   K   K   M   M   E   V   Q   K   K   S   F   E   E   K   Y
gaa gtc ttc atg gat gtt tgc caa aat ttt caa cca gtt ttc cgt tac ttc tgc atg gaa
 E   V   F   M   D   V   C   Q   N   F   Q   P   V   F   R   Y   F   C   M   E
aaa ttc ttg gat cca gct att tgg ttt gag aag cga ttg gct tat acg cgc agt gta gct
 K   F   L   D   P   A   I   W   F   E   K   R   L   A   Y   T   R   S   V   A
act tct tct att gtt ggt tac ata ctt gga ctt ggt gat aga cat gta cag aat atc ttg
 T   S   S   I   V   G   Y   I   L   G   L   G   D   R   H   V   Q   N   I   L
ata aat gag cag tca gca gaa ctt gta cat ata gat cta ggt gtt gct ttt gaa cag ggc
 I   N   E   Q   S   A   E   L   V   H   I   D   L   G   V   A   F   E   Q   G
aaa atc ctt cct act cct gag aca gtt cct ttt aga ctc acc aga gat att gtg gat ggc
 K   I   L   P   T   P   E   T   V   P   F   R   L   T   R   D   I   V   D   G
atg ggc att acg ggt gtt gaa ggt gtc ttc aga aga tgc tgt gag aaa acc atg gaa gtg
```

```
 M   G   I   T   G   V   E   G   V   F   R   R   C   C   E   K   T   M   E   V
atg aga aac tct cag gaa act ctg tta acc att gta gag gtc ctt cta tat gat cca ctc
 M   R   N   S   Q   E   T   L   L   T   I   V   E   V   L   L   Y   D   P   L
ttt gac tgg acc atg aat cct ttg aaa gct ttg tat tta cag cag agg ccg gaa gat gaa
 F   D   W   T   M   N   P   L   K   A   L   Y   L   Q   Q   R   P   E   D   E
act gag ctt cac cct act ctg aat gca gat gac caa gaa tgc aaa cga aat ctc agt gat
 T   E   L   H   P   T   L   N   A   D   D   Q   E   C   K   R   N   L   S   D
att gac cag agt ttc aac aaa gta gct gaa cgt gtc tta atg aga cta caa gag aaa ctg
 I   D   Q   S   F   N   K   V   A   E   R   V   L   M   R   L   Q   E   K   L
aaa gga gtg gaa gaa ggc act gtg ctc agt gtt ggt gga caa gtg aat ttg ctc ata cag
 K   G   V   E   E   G   T   V   L   S   V   G   G   Q   V   N   L   L   I   Q
cag gcc ata gac ccc aaa aat ctc agc cga ctt ttc cca gga tgg aaa gct tgg gtg tga
 Q   A   I   D   P   K   N   L   S   R   L   F   P   G   W   K   A   W   V   -
```

ATM SINT:

```
atg agt cta gta ctt aat gat ctg ctt atc tgc tgc cgt caa cta gaa cat gat aga gct
 M   S   L   V   L   N   D   L   L   I   C   C   R   Q   L   E   H   D   R   A
aca gaa cga aag aaa gaa gtt gag aaa ttt aag cgc ctg att cga gat cct gaa aca att
 T   E   R   K   K   E   V   E   K   F   K   R   L   I   R   D   P   E   T   I
aaa cat cta gat cgg cat tca gat tcc aaa caa gga aaa tat ttg aat tgg gat gct gtt
 K   H   L   D   R   H   S   D   S   K   Q   G   K   Y   L   N   W   D   A   V
ttt aga ttt tta cag aaa tat att cag aaa gaa aca gaa tgt ctg aga ata gca aaa cca
 F   R   F   L   Q   K   Y   I   Q   K   E   T   E   C   L   R   I   A   K   P
aat gta tca gcc tca aca caa gcc tcc agg cag aaa aag atg cag gaa atc agt agt ttg
 N   V   S   A   S   T   Q   A   S   R   Q   K   K   M   Q   E   I   S   S   L
gtc aaa tac ttc atc aaa tgt gca aac aga aga gca cct agg cta aaa tgt caa gaa ctc
 V   K   Y   F   I   K   C   A   N   R   R   A   P   R   L   K   C   Q   E   L
tta aat tat atc atg gat aca gtg aaa gat tca tct aat ggt gct att tac gga gct gat
 L   N   Y   I   M   D   T   V   K   D   S   S   N   G   A   I   Y   G   A   D
tgt agc aac ata cta ctc aaa gac att ctt tct gtg aga aaa tac tgg tgt gaa ata tct
 C   S   N   I   L   L   K   D   I   L   S   V   R   K   Y   W   C   E   I   S
cag caa cag tgg tta act ttt gga tat aga cgt tta gaa gac ttt atg gca tct cat tta
 Q   Q   Q   W   L   T   F   G   Y   R   R   L   E   D   F   M   A   S   H   L
gat tat ctg gtt ttg gaa tgg cta aat ctt caa gat act gaa tac aac tta tct tct ttt
 D   Y   L   V   L   E   W   L   N   L   Q   D   T   E   Y   N   L   S   S   F
cct ttt att tta tta aac tac aca aat att gag gat ttc tat aga tct tgt tat aag gtt
 P   F   I   L   L   N   Y   T   N   I   E   D   F   Y   R   S   C   Y   K   V
tgg tgt cct gaa cta gaa gaa ctt cat tac caa gca gca tgg agg aat atg cag tgg gac
 W   C   P   E   L   E   E   L   H   Y   Q   A   A   W   R   N   M   Q   W   D
cat tgc act tcc gtc agc aaa gaa gta gaa gga acc agt tac cat gaa tca ttg tac aat
 H   C   T   S   V   S   K   E   V   E   G   T   S   Y   H   E   S   L   Y   N
gct cta caa tct cta aga gac aga gaa ttc tct aca ttt tat gaa agt ctc aaa tat gcc
 A   L   Q   S   L   R   D   R   E   F   S   T   F   Y   E   S   L   K   Y   A
aga gta aaa gaa gtg gaa gag atg tgt aag cgc agc ctt gag tct gtg tat tcg ctc tat
 R   V   K   E   V   E   E   M   C   K   R   S   L   E   S   V   Y   S   L   Y
ccc aca ctt agc agg ttg cag gcc att gga gag ctg gaa agc att ggg gag ctt ttc tca
 P   T   L   S   R   L   Q   A   I   G   E   L   E   S   I   G   E   L   F   S
aga tca gtc aca cat aga caa ctc tct gaa gta tat att aag tgg cag aaa cac tcc cag
 R   S   V   T   H   R   Q   L   S   E   V   Y   I   K   W   Q   K   H   S   Q
ctt ctc aag gac agt gat ttt agt ttt cag gag cct atc atg gct cta cgc aca gtc att
 L   L   K   D   S   D   F   S   F   Q   E   P   I   M   A   L   R   T   V   I
ttg gag atc ctg atg gaa aag gaa atg gac aac tca caa aga gaa tgt att aag gac att
 L   E   I   L   M   E   K   E   M   D   N   S   Q   R   E   C   I   K   D   I
ctc acc aaa cac ctt gta gaa ctc tct ata ctg gcc aga act ttc aag aac act cag ctc
 L   T   K   H   L   V   E   L   S   I   L   A   R   T   F   K   N   T   Q   L
cct gaa agg gca ata ttt caa att aaa cag tac aat tca gtt agc tgt gga gtc tct gag
```

P E R A I F Q I K Q Y N S V S C G V S E
tgg cag ctg gaa gaa gca caa gta ttc tgg gca aaa aag gag cag agt ctt gcc ctg agt
W Q L E E A Q V F W A K K E Q S L A L S
att ctc aag caa atg atc aag aag ttg gat gcc agc tgt gca gcg aac aat ccc agc cta
I L K Q M I K K L D A S C A A N N P S L
aaa ctt aca tac aca gaa tgt ctg agg gtt tgt ggc aac tgg tta gca gaa acg tgc tta
K L T Y T E C L R V C G N W L A E T C L
gaa aat cct gcg gtc atc atg cag acc tat cta gaa aag gca gta gaa gtt gct gga aat
E N P A V I M Q T Y L E K A V E V A G N
tat gat gga gaa agt agt gat gag cta aga aat gga aaa atg aag gca ttt ctc tca tta
Y D G E S S D E L R N G K M K A F L S L
gcc cgg ttt tca gat act caa tac caa aga att gaa aac tac atg aaa tca tcg gaa ttt
A R F S D T Q Y Q R I E N Y M K S S E F
gaa aac aag caa gct ctc ctg aaa aga gcc aaa gag gaa gta ggt ctc ctt agg gaa cat
E N K Q A L L K R A K E E V G L L R E H
aaa att cag aca aac aga tac aca gta aag gtt cag cga gag ctg gag ttg gat gaa tta
K I Q T N R Y T V K V Q R E L E L D E L
gcc ctg cgt gca ctg aaa gag gat cgt aaa cgc ttc tta tgt aaa gca gtt gaa aat tat
A L R A L K E D R K R F L C K A V E N Y
atc aac tgc tta tta agt gga gaa gaa cat gat atg tgg gta ttc cga ctt tgt tcc ctc
I N C L L S G E E H D M W V F R L C S L
tgg ctt gaa aat tct gga cag ctt gtt aag ggc cgt gat gac ctg aga caa gat gct gtc
W L E N S G Q L V K G R D D L R Q D A V
atg caa cag gtc ttc cag atg tgt aat aca tta ctg cag aga aac acg gaa act agg aag
M Q Q V F Q M C N T L L Q R N T E T R K
agg aaa tta act atc tgt act tat aag gtg gtt ccc ctc tct cag cga agt ggt gtt ctt
R K L T I C T Y K V V P L S Q R S G V L
gaa tgg tgc aca gga act gtc ccc att ggt gaa ttt ctt gtt aac aat gaa gat ggt gct
E W C T G T V P I G E F L V N N E D G A
cat aaa aga tac agg cca aat gat ttc agt gcc ttt cag tgc caa aag aaa atg atg gag
H K R Y R P N D F S A F Q C Q K K M M E
gtg caa aaa aag tct ttt gaa gag aaa tat gaa gtc ttc atg gat gtt tgc caa aat ttt
V Q K K S F E E K Y E V F M D V C Q N F
caa cca gtt ttc cgt tac ttc tgc atg gaa aaa ttc ttg gat cca gct att tgg ttt gag
Q P V F R Y F C M E K F L D P A I W F E
aag cga ttg gct tat acg cgc agt gta gct act tct tct att gtt ggt tac ata ctt gga
K R L A Y T R S V A T S S I V G Y I L G
ctt ggt gat aga cat gta cag aat atc ttg ata aat gag cag tca gca gaa ctt gta cat
L G D R H V Q N I L I N E Q S A E L V H
ata gat cta ggt gtt gct ttt gaa cag ggc aaa atc ctt cct act cct gag aca gtt cct
I D L G V A F E Q G K I L P T P E T V P
ttt aga ctc acc aga gat att gtg gat ggc atg ggc att acg ggt gtt gaa ggt gtc ttc
F R L T R D I V D G M G I T G V E G V F
aga aga tgc tgt gag aaa acc atg gaa gtg atg aga aac tct cag gaa act ctg tta acc
R R C C E K T M E V M R N S Q E T L L T
att gta gag gtc ctt cta tat gat cca ctc ttt gac tgg acc atg aat cct ttg aaa gct
I V E V L L Y D P L F D W T M N P L K A
ttg tat tta cag cag agg ccg gaa gat gaa act gag ctt cac cct act ctg aat gca gat
L Y L Q Q R P E D E T E L H P T L N A D
gac caa gaa tgc aaa cga aat ctc agt gat att gac cag agt ttc aac aaa gta gct gaa
D Q E C K R N L S D I D Q S F N K V A E
cgt gtc tta atg aga cta caa gag aaa ctg aaa gga gtg gaa gaa ggc act gtg ctc agt
R V L M R L Q E K L K G V E E G T V L S
gtt ggt gga caa gtg aat ttg ctc ata cag cag gcc ata gac ccc aaa aat ctc agc cga
V G G Q V N L L I Q Q A I D P K N L S R
ctt ttc cca gga tgg aaa gct tgg gtg tga
L F P G W K A W V -

Fig. S1 c-DNA sequences of ATM 3-52 and ATM 4-53 originated from canonical splicing of the ATM mRNA, identified in vivo in the blood of AT patients treated with intra-erythrocyte Dexamethasone (EryDex) in a phase II Clinical trial [1].

ATM SINT was in silico designed with the addition of further functional domains. The ORF prediction is shown under the nucleotide sequence. The translation could start from the native starting codon for ATM 4-53 and ATM SINT, whilst ATM 3-52 translation starts 714 bp upstream than the miniATM starting codon, rather than at the native starting codon

miniATM: published by Menotta et al. [2].

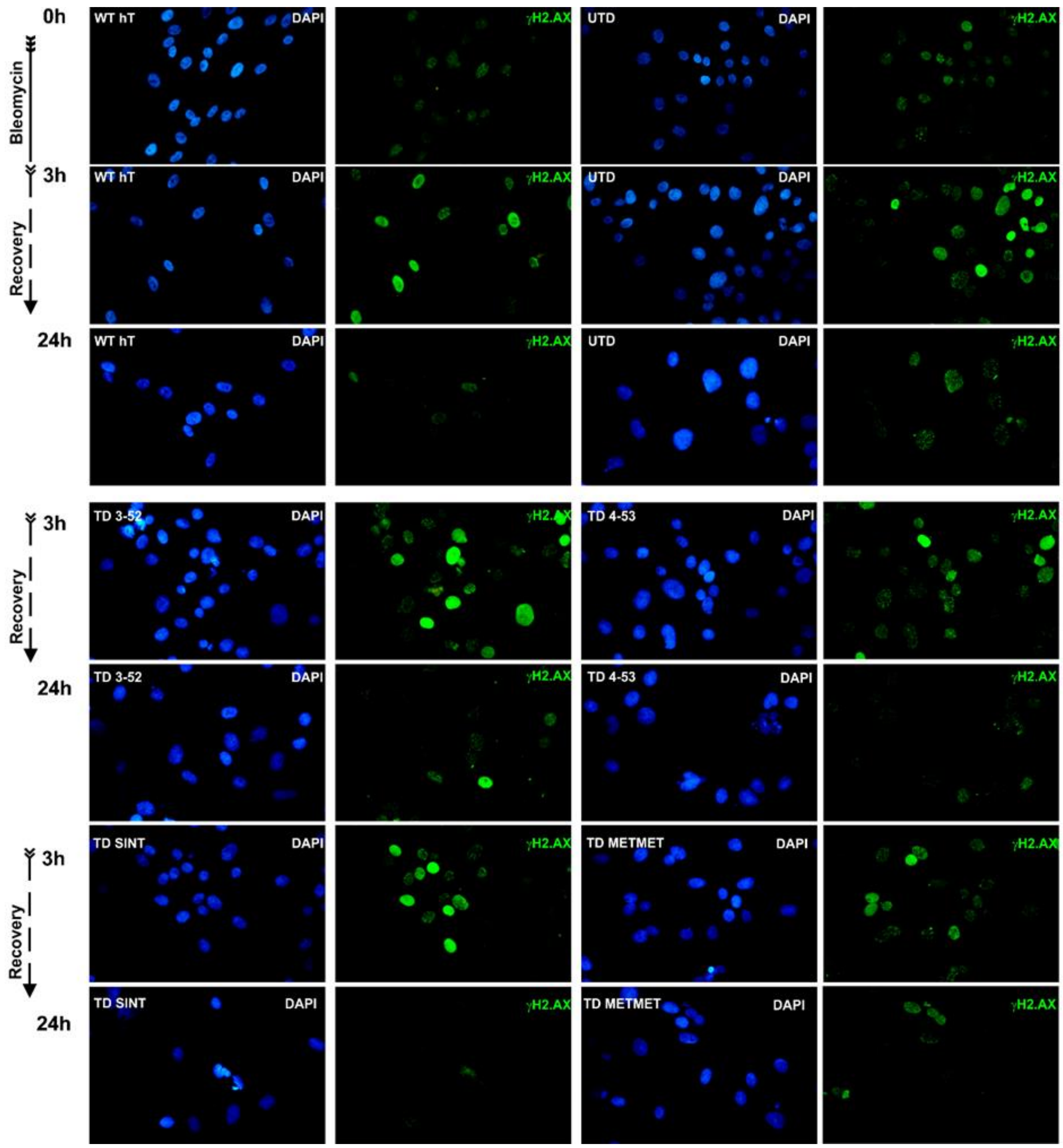

Fig. S2 Typical images of γH2AX IF experiments of WT hT and AT 648 hT transduced and untransduced cells, treated with bleomycin for 3h and then kept in recovery course for 24h. Quantification of γH2AX is reported in Fig. 2. In all AT 648 hT transduced cells there was an increase of γH2AX after 3h bleomycin treatment compared to UTD, and a reduction after 24h of recovery

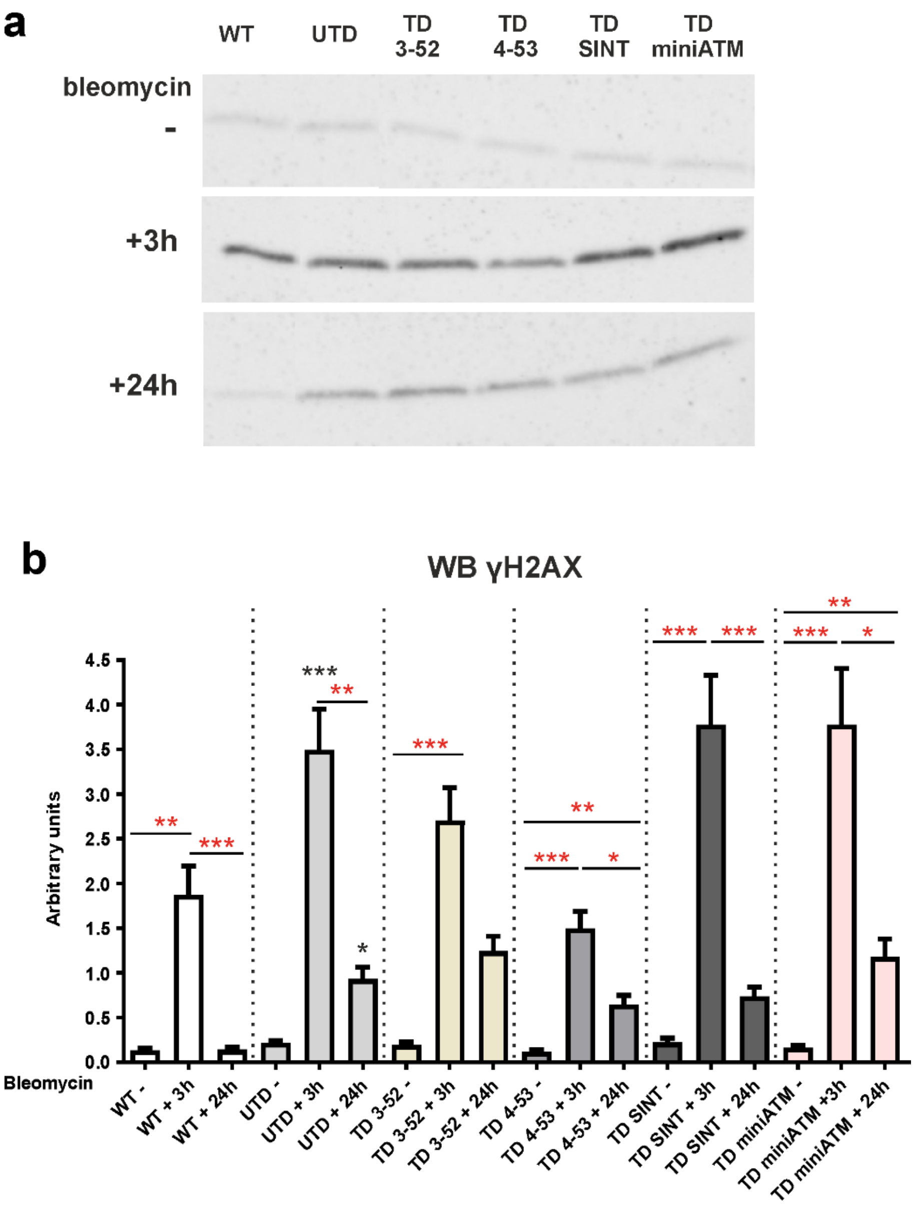

Fig. S3 WB quantification of γH2AX confirms IF analyses. a) Representative western blot and b) quantification of γH2AX of WT hT and AT 648 hT transduced and untransduced cells, treated with bleomycin for 3h and kept in recovery for 24 hours. In WT hT cells and TD cells there was an increase of γH2AX after 3h of bleomycin treatment compared to their basal counterparts, while there was a reduction of γH2AX after 24h of recovery in the same cells, except for TD 3-52. Only TD 3-52 indeed, did not significantly decrease the phosphorylation of H2AX upon 24h recovery. Similarly, UTD showed an enhanced phosphorylation of H2AX after 3h of bleomycin and a H2AX dephosphorylation after the recovery condition. It is to be noted that WB method does not monitor individual nuclei analysis. IF quantification is required instead to do this (Fig. 2-3). Red asterisks refer to intra sample comparison, while the black ones refer to statistical comparison with the control cell line UTD- (Friedman test followed by Dunn's test). Graphs show Mean with SEM n= 9

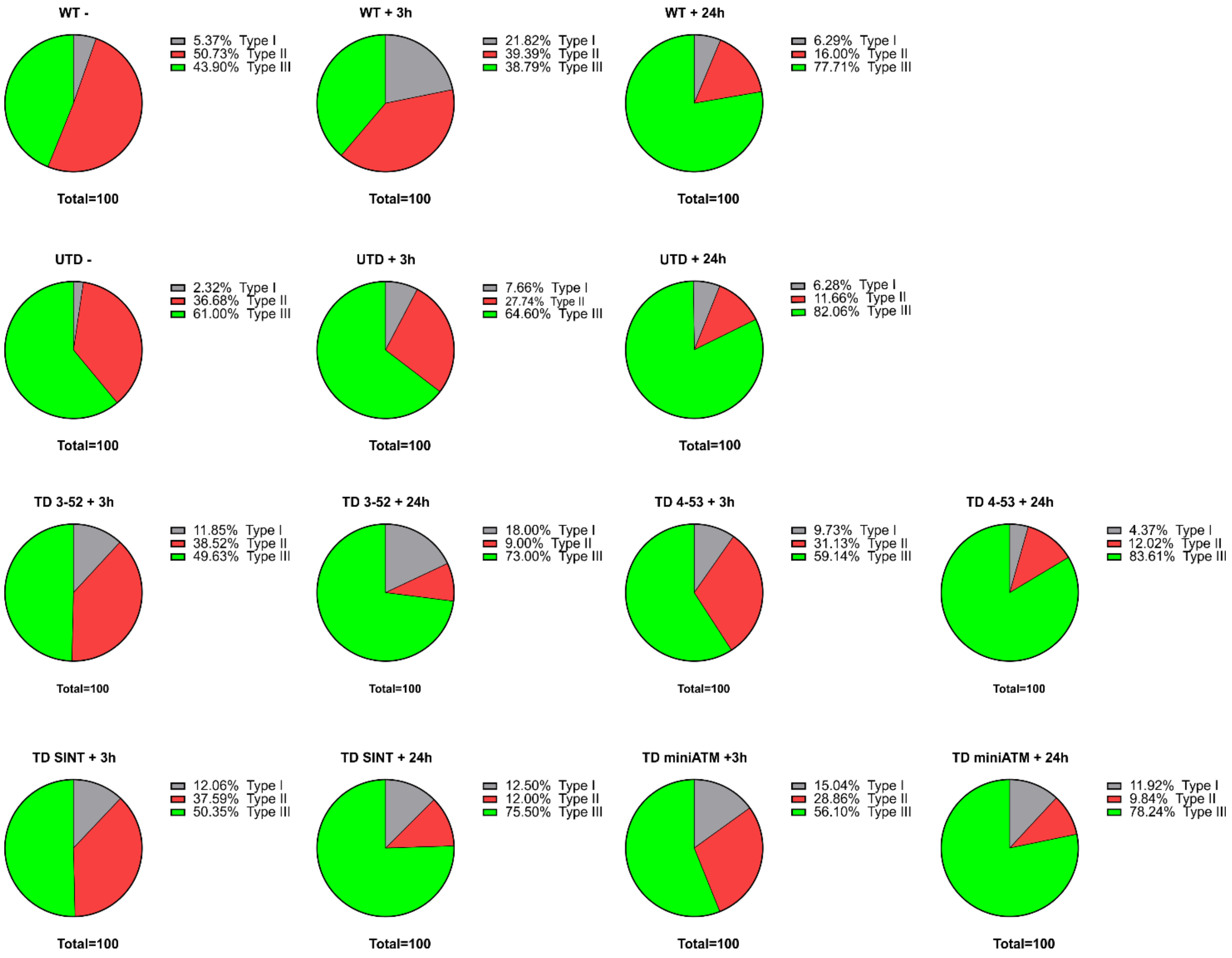

Fig. S4 Foci type staining of each tested cell line after each treatment condition. WT hT presented an enhanced Type I after bleomycin treatment, and an increase of Type III with a diminution of Type I after 24h of recovery. All TD cells improved Type I stain pattern after 3h of bleomycin treatment compared to UTD at basal condition. In contrast, UTD have a slight increase of Type I staining pattern in comparison with TD cells over 3h of drug treatment, due to the lack of an active ATM. After 24h of bleomycin, Type III staining type boosted in all the tested cell lines, despite different number of foci (See Type III quantification in Figure 3b)

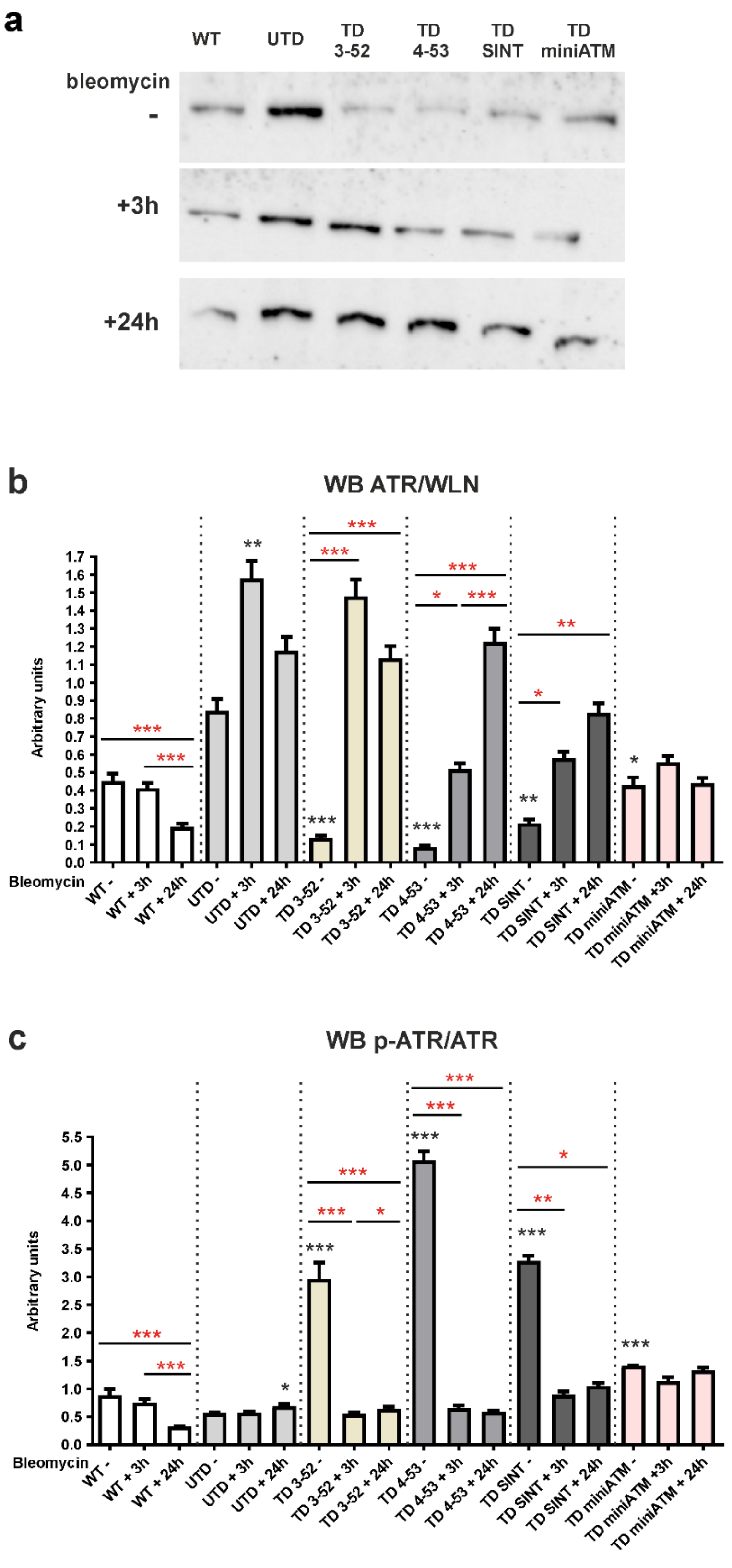

Fig. S5 H2AX phosphorylation is not affected by ATR in AT 648 hT transduced cell lines. a) ATR representative western blot and b) quantification of ATR/WLN and c) of p-ATR/ATR WB experiments of WT hT and AT 648 hT transduced and untransduced cells, treated with bleomycin for 3h and kept in recovery for 24 hours. UTD displayed a tiny quantity of p-ATR/ATR ratio in all treatment conditions, as the amount of ATR was constantly elevated in these cell lines, and even higher after 3h of drug treatment than their basal condition. In UTD under 24h of recovery, total ATR did not change

while p-ATR/ATR enhanced. In contrast, TD cells have a higher ratio of p-ATR/ATR at basal condition due to the lower amount of ATR total protein, compared to UTD. Upon bleomycin treatment, the increment of total ATR quantity was observed in TD 3-52, TD 4-53 and TD SINT, with a concomitant decrease of p-ATR/ATR ratio. Upon recovery condition, TD 4-53 and TD SINT increased total ATR with a reduction of pATR/ATR compared to their untreated counterparts. In contrast, TD 3-52 decrease the amount of ATR with a concomitant reduction of pATR/ATR than their basal condition. TD miniATM did not change ATR protein amounts and p-ATR/ATR ratio after drug treatment and after the recovery condition. In WT hT treated cells, total ATR and pATR/ATR ratio did not differ than basal condition, while both targets decreased after 24h recovery. Red asterisks refer to intra sample comparison, while the black ones refer to the comparison with the UTD- (Friedman test followed by Dunn’s test). Graphs show Mean with SEM, n = 6

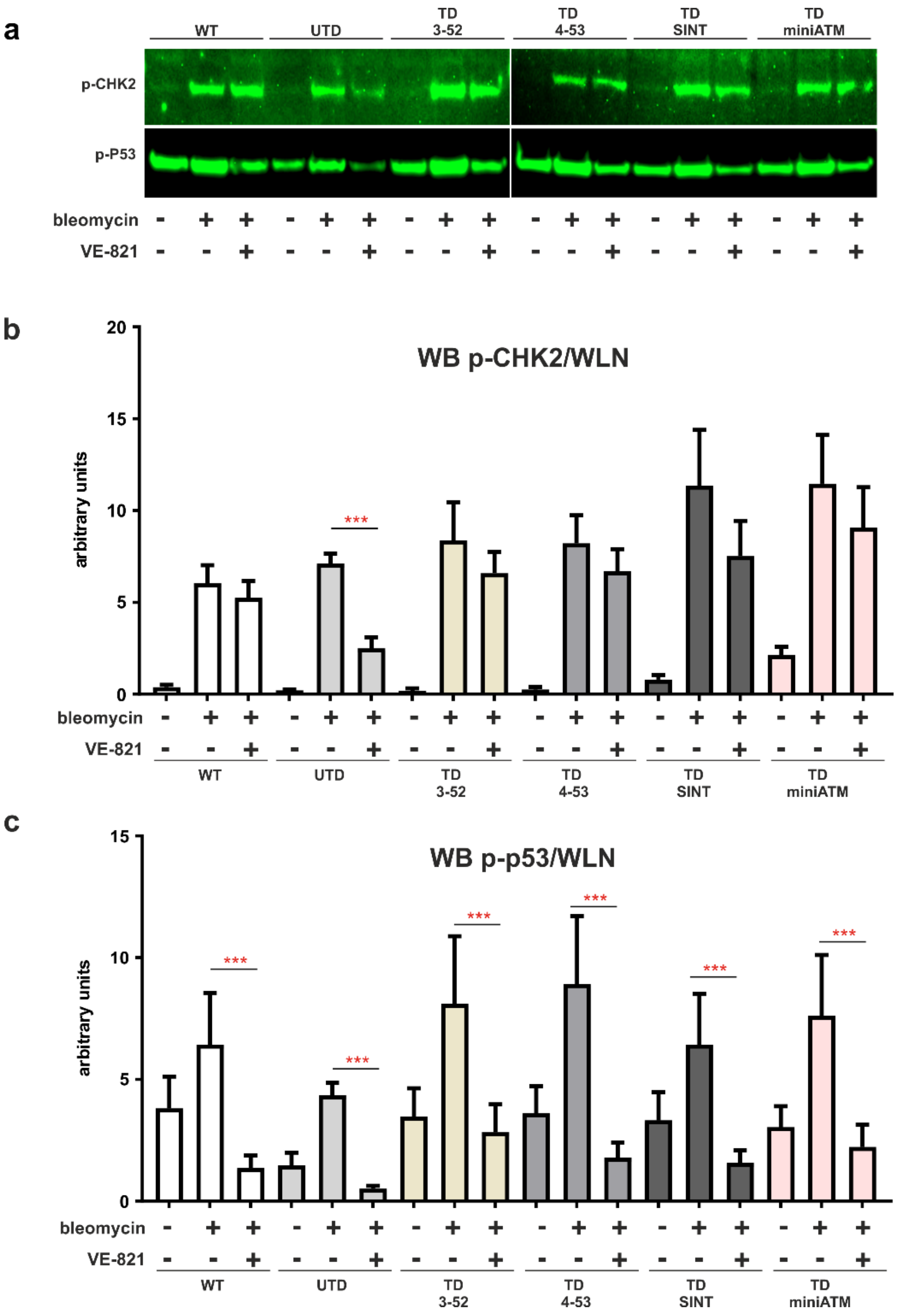

Fig. S6 CHK2 and p53 phosphorylation is induced by ATR in UTD. a) p-CHK2 and p-p53 representative western blots and b) quantification of p-CHK2/WLN and c) of p-p53/WLN WB experiments of WT hT and AT 648 hT transduced and untransduced cells, pre-treated with VE-821 (ATR kinase inhibitor) for 1h and then co-exposed with bleomycin and VE-821 for 3h. WT hT cells and all TD cells did not show any significant changes in p-CHK2 when VE-821 is added to the cells, reinforcing the activity of ATM variants in phosphorylating the cell cycle checkpoint CHK2. On the contrary, UTD presented a reduced phosphorylation of CHK2 in the presence of VE-821, confirming the role of ATR kinase in phosphorylating CHK2 in these cells.

p53, instead displayed a decrease of its phosphorylation in all tested cells when VE-821 is added in combination with bleomycin compared to the cells treated with bleomycin alone, even though the reduction is much more evident in UTD. TD cells behaved as WT hT cells.

Red asterisks refer to intra sample comparison (Wilcoxon test). Graphs show Mean with SEM, n = 5

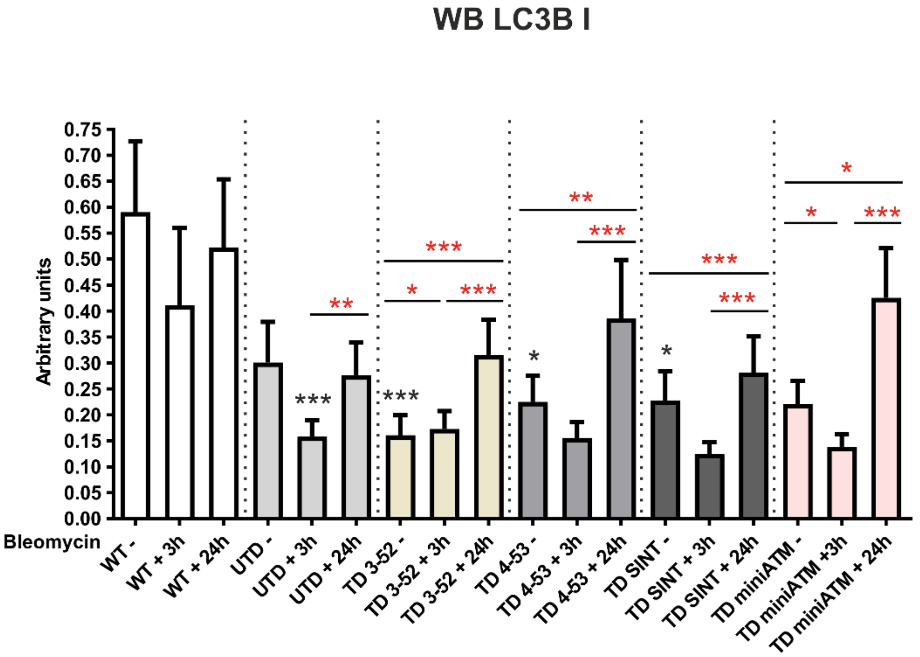

Fig. S7 UTD present autophagy impairment. Quantification of immune reactive band of LC3B-I in all tested cell lines, treated with bleomycin for 3h and then kept in recovery course for 24 hours. LC3B-I accumulation was noticed in UTD at basal condition, compared to TD cells (except for TD miniATM) suggesting a lower conversion of LC3B-I to the lipidated form LC3B-II in UTD. When bleomycin was added, a reduced quantity of LC3B-I was found in UTD (but with a higher LC3B II/I ratio, see Fig. 7), TD 4-53, TD SINT (although not statistically significant) and in TD miniATM, reinforcing the results obtained from LC3B II/I analysis (see LC3B II/I quantification in Fig. 7). TD 3-52 enhanced LC3B-I after 3h of bleomycin treatment. 24h post drug treatment a recovery of LC3B-I protein was found in all TD cells and UTD. In contrast, WT hT cells did not change LC3B-I amount during treatment. Red asterisks refer to intra sample comparison, while the black ones refer to the comparison with the UTD- (Friedman test followed by Dunn's test). Graphs show Mean with SEM, n = 7

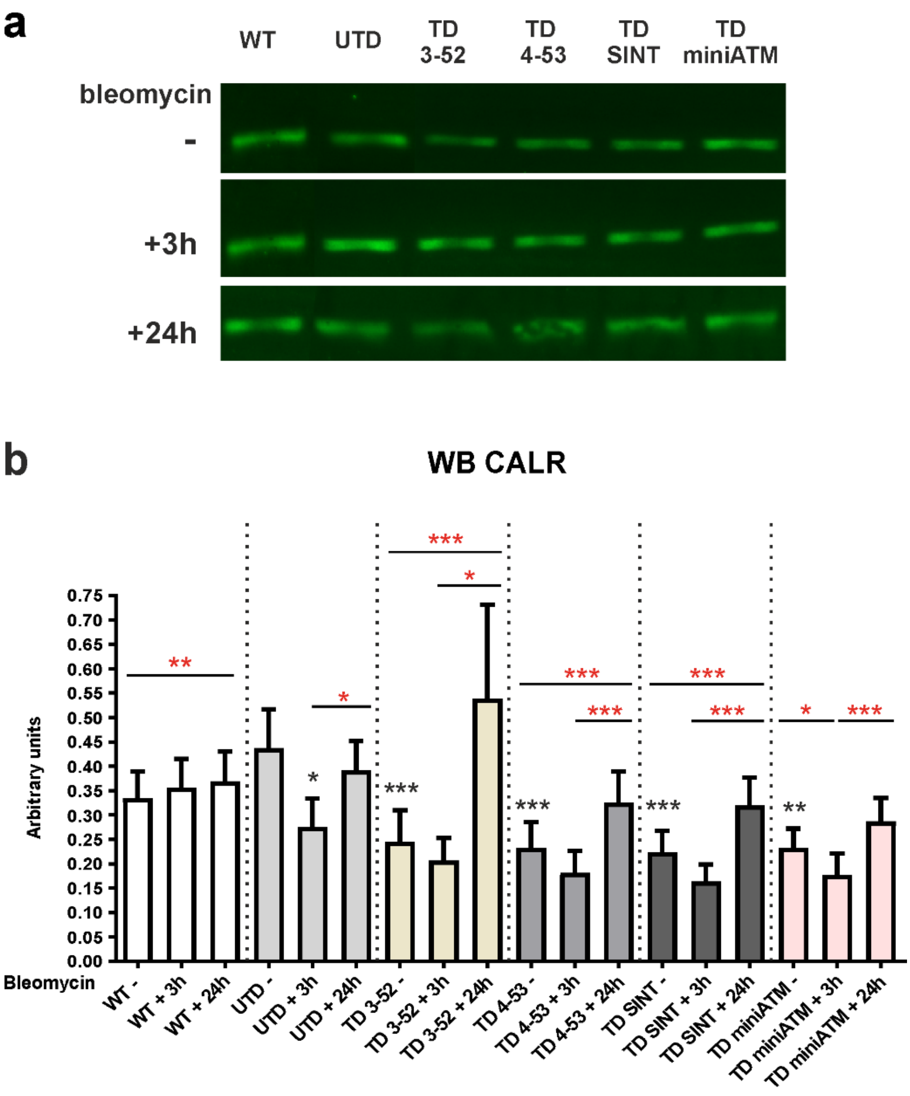

Fig. S8 ER stress is a characteristic in UTD. a) Representative image and b) quantification from WB of CALR expression in all tested cell lines, treated with bleomycin for 3h and then kept in recovery course for 24h. At basal condition the amount of CALR was higher in UTD than TD cells, indicating ER stress when ATM is not present. When bleomycin is added, CALR statistically decrease only in UTD and TD miniATM, even though a reduction was observed in all TD cells, followed by an enhancement of its amount in the recovery condition in all the tested cell lines. Also in this case, CALR amount is not affected by drug treatment in WT hT cells, whereas it is enhanced 24h post bleomycin compared to their basal counterparts. Red asterisks refer to intra sample comparison, while the black ones refer to the comparison with the UTD- (Friedman test followed by Dunn test). Graphs show Mean with SEM, n = 7

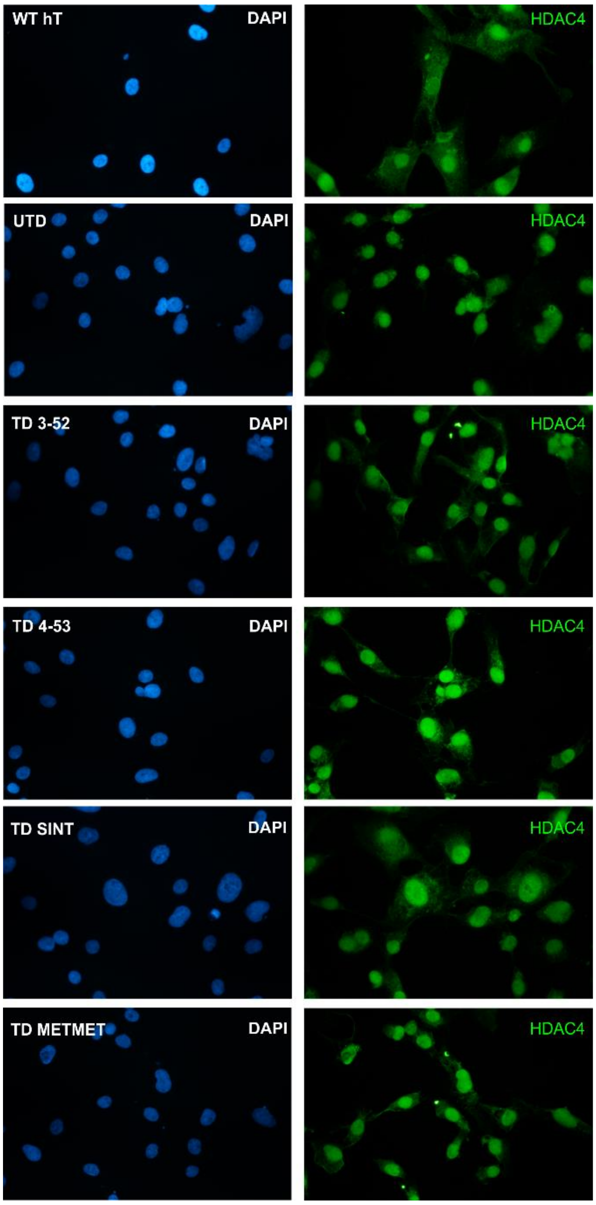

Fig. S9 Typical images illustrating the nuclear localization of HDAC4 in all tested cell lines stained by IF in WT hT and AT 648 hT transduced and untransduced cells. Quantification of the nuclear HDAC4 levels is reported in Fig. 11. All AT 648 hT transduced cells could alter HDAC4 nuclear/cytosol shuttle, showing less accumulation of nuclear HDAC4 in these cells, compared to UTD

References

1. Menotta M, Biagiotti S, Spapperi C, Orazi S, Rossi L, Chessa L, Leuzzi V, D'Agnano D, Soresina A, Micheli R, et al. (2017) ATM splicing variants as biomarkers for low dose dexamethasone treatment of A-T. Orphanet J Rare Dis 12:126. 10.1186/s13023-017-0669-2
2. Menotta M, Biagiotti S, Bianchi M, Chessa L, and Magnani M (2012) Dexamethasone partially rescues ataxia telangiectasia-mutated (ATM) deficiency in ataxia telangiectasia by promoting a shortened protein variant retaining kinase activity. J Biol Chem 287:41352-41363. 10.1074/jbc.M112.344473

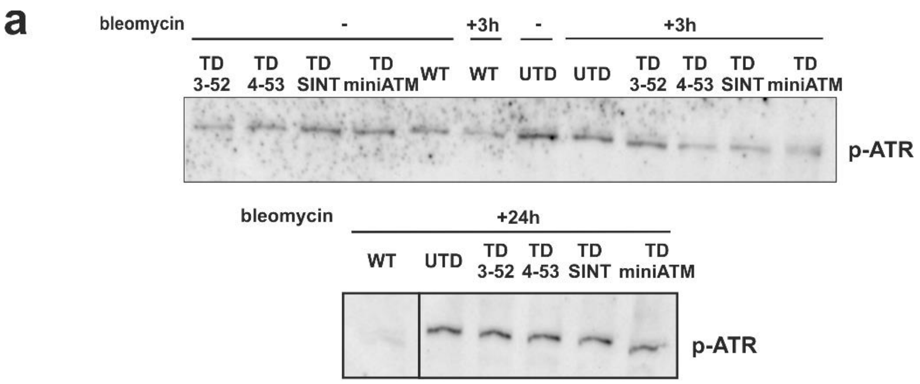

Original Fig. 4a

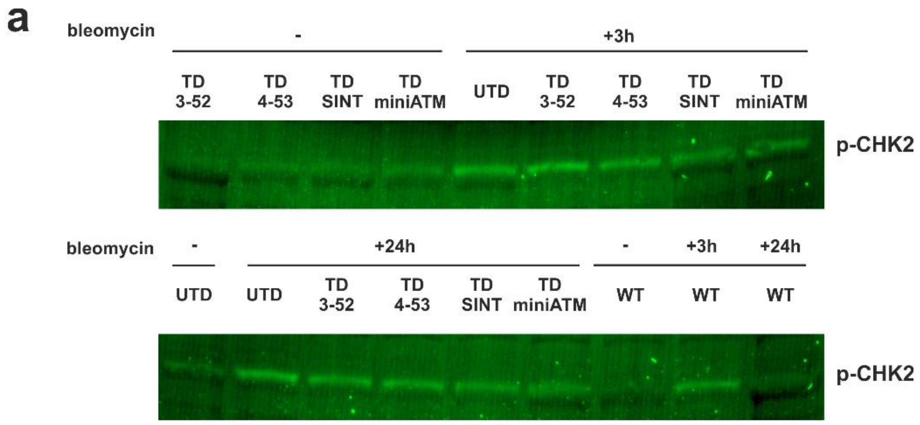

Original Fig. 5a

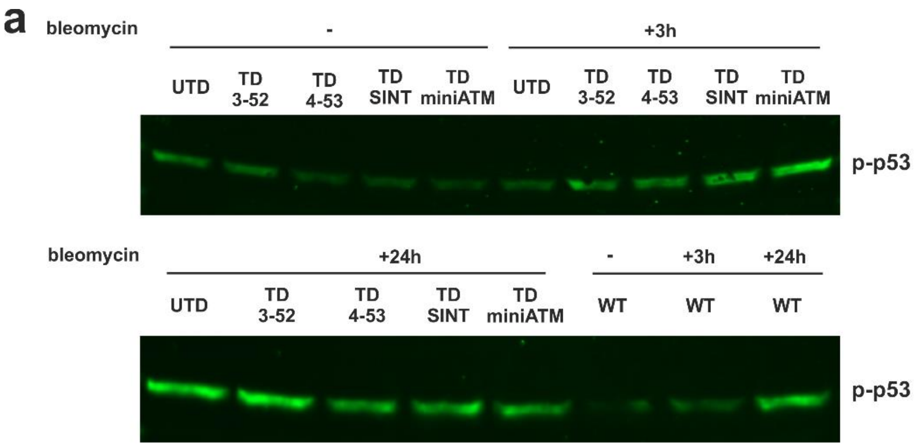

Original Fig. 6a

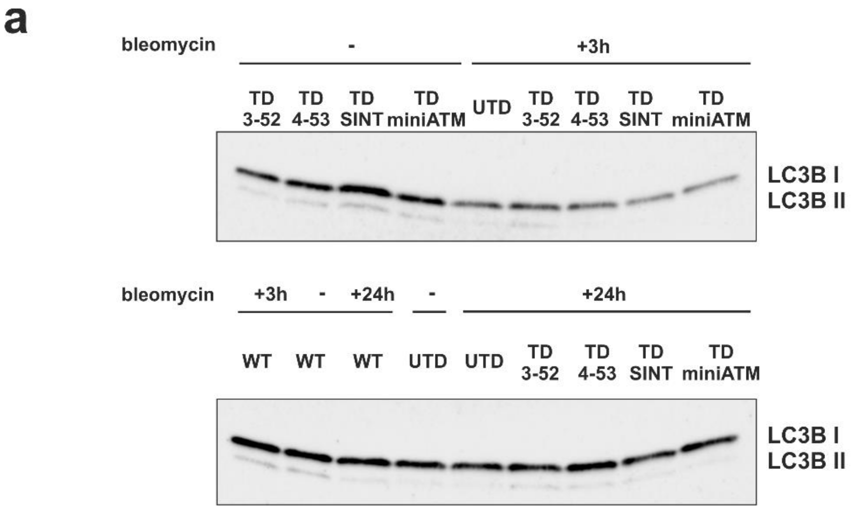

Original Fig. 7a

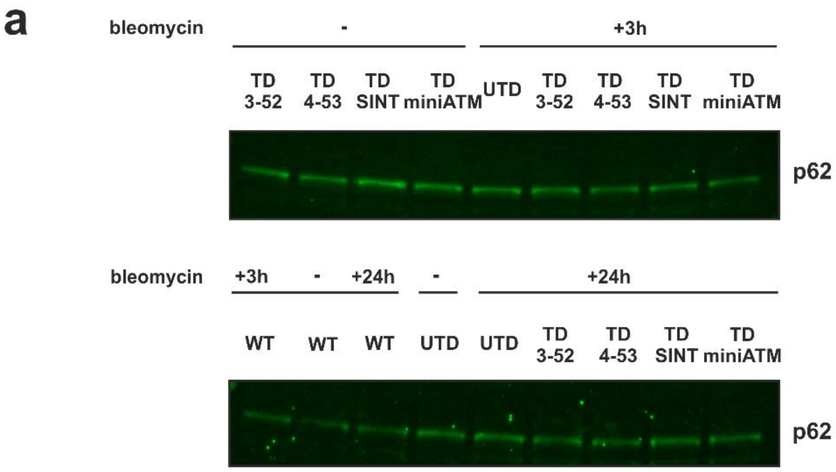

Original Fig. 8a

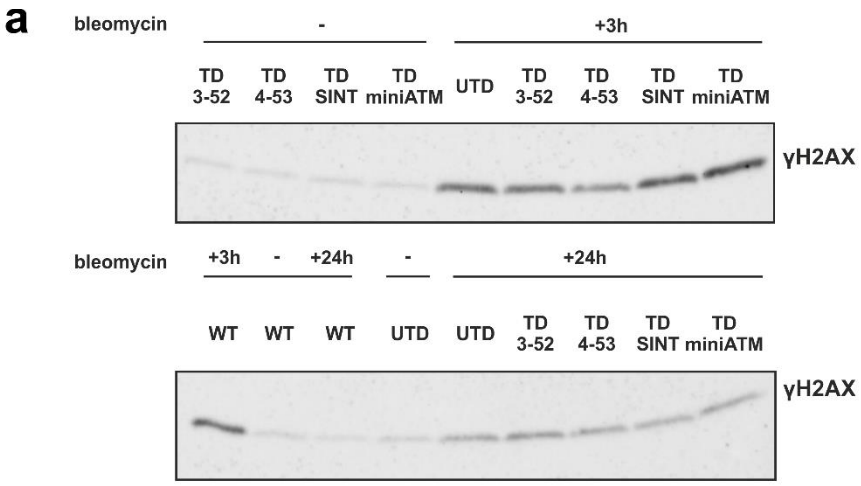

Original Fig. S3a

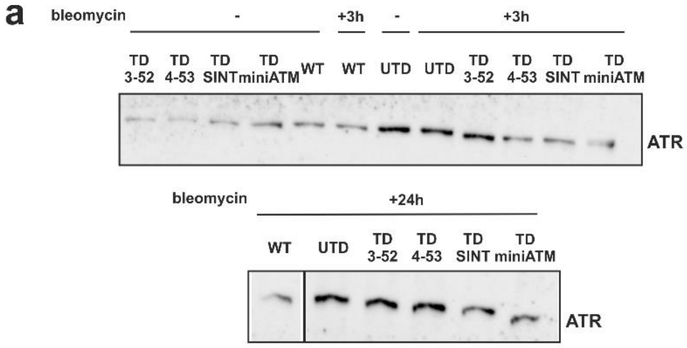

Original Fig. S5a

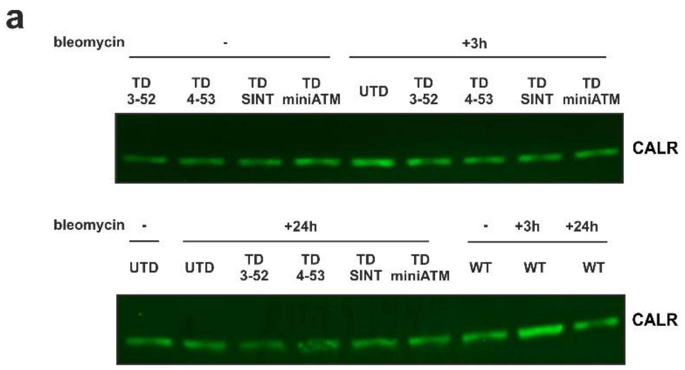

Original Fig. S8a
